# Supplementary material for: In-depth human plasma proteome analysis captures tissue proteins and transfer of protein variants across the placenta
Source: eLife. 2019 Apr 8;8:e41608. doi: 10.7554/eLife.41608 (PMC6519984; doi:10.7554/eLife.41608)
Supplement: Supplementary file 1. — 1% FDR cutoff at PSM, peptide and protein level, MSGF +with Percolator processing. Label-free, protein centric analysis. * Spiked amount was 4 ng/mL prior to depletion. NA indicates that PSA was not spiked into the sample. ** Percentage of proteins identified by a single peptide, *** Percentage of PSMs originating from the highest abundant protein. [file elife-41608-supp1.docx]

| **Experiment** | **HiRIEF pH range** | **Load (mg)** | **# Proteins** | **# Peptides** | **# PSMs** | **Spiked* PSA detected?** | **%1-peptide ID´s**** | **% PSM APOB***** |
| --- | --- | --- | --- | --- | --- | --- | --- | --- |
| MS runtime control (72 repeated MS injections) | NA |  | 241 | 2588 | 148304 | no | 30,3 | 6,5 |
| 1 | 3.0-10.0 | 1.0 | 1626 | 15424 | 123262 | NA | 30,0 | 10,1 |
| 2 |  | 0.6 | 1791 | 16372 | 118983 | no | 27,2 | 8,2 |
| 3 | 3.7-4.9 | 1.0 | 1517 | 8566 | 72287 | NA | 31,4 | 9,3 |
| 4 | 3.7-4.05 | 0.2 | 1068 | 3230 | 25033 | no | 47,4 | 7,1 |
| 5 |  | 0.6 | 1394 | 3871 | 25472 | no | 51,4 | 6,6 |
| 6 |  | 1.0 | 1774 | 5421 | 39351 | no | 47,7 | 6,8 |
| 7 | 4.0-4.25 | 0.2 | 904 | 2916 | 29224 | yes, 2 PSM | 45,6 | 7,1 |
| 8 |  | 0.6 | 1534 | 4629 | 47470 | yes, 2 PSM | 47,4 | 8,4 |
| 9 |  | 1.0 | 1888 | 6012 | 64970 | yes, 5 PSM | 46,2 | 7,8 |
| 10 |  | 1.0 | 1360 | 4695 | 46230 | NA | 44,1 | 7,8 |
| 11 |  | 2.0 | 1659 | 5324 | 53323 | NA | 46,2 | 6,9 |
| 12 |  | 4.0 | 1812 | 6339 | 71727 | NA | 43,8 | 8,2 |
| 13 |  | 6.0 | 1608 | 6149 | 70376 | NA | 42,8 | 10,7 |
| 14 | 4.2-4.45 | 0.2 | 913 | 2992 | 30067 | no | 47,9 | 9,5 |
| 15 |  | 0.6 | 1422 | 4470 | 41619 | no | 48,9 | 9,4 |
| 16 |  | 1.0 | 1803 | 6042 | 63329 | no | 45,0 | 9,1 |

**Supplementary file 1.** Summary of the number of identifications from the optimization experiment.  1% FDR cutoff at PSM, peptide and protein level, MSGF+ with Percolator processing. Label-free, protein centric analysis. * Spiked amount was 4ng/mL prior to depletion. NA indicates that PSA was not spiked into the sample. ** Percentage of proteins identified by a single peptide, *** Percentage of PSMs originating from the highest abundant protein.
