# Supplementary material for: In-depth human plasma proteome analysis captures tissue proteins and transfer of protein variants across the placenta
Source: eLife. 2019 Apr 8;8:e41608. doi: 10.7554/eLife.41608 (PMC6519984; doi:10.7554/eLife.41608)
Supplement: Supplementary file 2. — Search engines used were Andromeda (MaxQuant) (FDR 1%, PSM and protein level) and MSGF +and Percolator (PSM, peptide and protein level FDR 1%), Protein centric. [file elife-41608-supp2.docx]

|  |  |  | **MSGF+ and Percolator** | | | **Andromeda (MaxQuant)** | | | |
| --- | --- | --- | --- | --- | --- | --- | --- | --- | --- |
| **Experiment** | **HiRIEF range (pH)** | **Load (mg)** | **#Proteins** | **#Peptides (Unique Groups)** | **#PSMs** | **#Phos-peptides*** | **%PhosPeptides** | **#Phospho Proteins** | **%Phospho proteins** |
| MS runtime control | NA |  | 196 | 2264 | 129770 | 17 | 0,7 | 7 | 3,6 |
| 1 | 3.0-10.0 | 1.0 | 1393 | 11957 | 92882 | 134 | 1,1 | 69 | 4,9 |
| 2 |  | 0.6 | 1659 | 12944 | 97324 | 162 | 1,2 | 89 | 5,3 |
| 3 | 3.7-4.9 | 1.0 | 1406 | 7045 | 58047 | 140 | 2,0 | 68 | 4,8 |
| 4 | 3.7-4.05 | 0.2 | 948 | 2317 | 15998 | 57 | 2,4 | 37 | 3,8 |
| 5 |  | 0.6 | 1224 | 2964 | 17704 | 104 | 3,4 | 66 | 5,3 |
| 6 |  | 1.0 | 1546 | 4172 | 28288 | 119 | 2,8 | 69 | 4,5 |
| 7 | 4.0-4.25 | 0.2 | 881 | 2176 | 18845 | 34 | 1,6 | 18 | 2,1 |
| 8 |  | 0.6 | 1313 | 3745 | 35211 | 75 | 2,0 | 40 | 3,0 |
| 9 |  | 1.0 | 1691 | 4819 | 47569 | 99 | 2,3 | 56 | 3,5 |
| 10 |  | 1.0 | 1187 | 3540 | 32078 | 69 | 1,9 | 29 | 2,6 |
| 11 |  | 2.0 | 1449 | 4264 | 39202 | 105 | 2,4 | 60 | 4,1 |
| 12 |  | 4.0 | 1566 | 4748 | 48837 | 89 | 1,8 | 49 | 3,1 |
| 13 |  | 6.0 | 1480 | 4775 | 46447 | 80 | 1,7 | 46 | 3,1 |
| 14 | 4.2-4.45 | 0.2 | 926 | 2272 | 18103 | 19 | 0,8 | 12 | 1,3 |
| 15 |  | 0.6 | 1233 | 3541 | 30501 | 46 | 1,3 | 24 | 2,0 |
| 16 |  | 1.0 | 1645 | 5014 | 48458 | 56 | 1,1 | 37 | 2,2 |

**Supplementary file 2.** Summary of the number of identifications from the optimization experiments, database search allowing for phospho-modifications. Search engines used were Andromeda (MaxQuant) (FDR 1%, PSM and protein level) and MSGF+ and Percolator (PSM, peptide and protein level FDR 1%), Protein centric.
