# Supplementary material for: In-depth human plasma proteome analysis captures tissue proteins and transfer of protein variants across the placenta
Source: eLife. 2019 Apr 8;8:e41608. doi: 10.7554/eLife.41608 (PMC6519984; doi:10.7554/eLife.41608)
Supplement: Supplementary file 3. — Phosphosite probability calculations were performed within the MaxQuant software. GeLC = gel based (SDS PAGE) separation and digestion. [file elife-41608-supp3.docx]

|  |  |  | **Phosphosites (S-T-Y)** | | | |  | **Phosphosites (S-T-Y), percentages** | | |
| --- | --- | --- | --- | --- | --- | --- | --- | --- | --- | --- |
|  | **#Phospho-proteins** | **%Phospho-proteins** | **pSer** | **pThr** | **pTyr** | **Total # sites** |  | **pSer** | **pThr** | **pTyr** |
| **MS run time control** | 7 | 3,6 | 15 | 11 | 2 | 28 |  | 54 | 39 | 7 |
| **HiRIEF 3.0-10.0** | 111 | 5,5 | 145 | 65 | 19 | 229 |  | 63 | 28 | 8 |
| **HiRIEF 3.7-4.9** | 68 | 4,8 | 70 | 25 | 18 | 114 |  | 61 | 22 | 16 |
| **HiRIEF 3.7-4.05** | 106 | 5,7 | 115 | 33 | 22 | 170 |  | 68 | 19 | 13 |
| **HiRIEF_4.0-4.25** | 121 | 4,6 | 165 | 104 | 55 | 324 |  | 51 | 32 | 17 |
| **HiRIEF_4.2-4.45** | 57 | 2,9 | 29 | 37 | 16 | 82 |  | 35 | 45 | 20 |
| **GeLC** | 102 | 7,3 | 571 | 410 | 111 | 1092 |  | 52 | 38 | 10 |

**Supplementary file 3**. Summary of phosphodata from the different HiRIEF strip ranges in absolute number and percent. Phosphosite probability calculations were performed within the MaxQuant software. GeLC = gel based (SDS PAGE) separation and digestion.
