# Supplementary material for: In-depth human plasma proteome analysis captures tissue proteins and transfer of protein variants across the placenta
Source: eLife. 2019 Apr 8;8:e41608. doi: 10.7554/eLife.41608 (PMC6519984; doi:10.7554/eLife.41608)
Supplement: Supplementary file 5. — The proteins were ranked from smallest to largest inter-individual variation based on their coefficient of variation. The top of the list (least varying) was enriched for proteins linked to humoral activation proteins and receptor activity. [file elife-41608-supp5.docx]

| **Process** |  |  |  |
| --- | --- | --- | --- |
| **GO term** | **Description** | **P-value** | **FDR q-value** |
| GO:0002920 | regulation of humoral immune response | 1.16E-7 | 8.34E-4 |
| GO:0007155 | cell adhesion | 2.51E-7 | 9.04E-4 |
| GO:0022610 | biological adhesion | 2.74E-7 | 6.58E-4 |
|  |  |  |  |
| **Function** |  |  |  |
| **GO term** | **Description** | **P-value** | **FDR q-value** |
| GO:0004872 | receptor activity | 1.5E-7 | 2.12E-4 |
| GO:0060089 | molecular transducer activity | 1.5E-7 | 1.06E-4 |
| GO:0019838 | growth factor binding | 4.53E-6 | 2.13E-3 |
|  |  |  |  |
| **Component** |  |  |  |
| **GO term** | **Description** | **P-value** | **FDR q-value** |
| GO:0016021 | integral component of membrane | 1.03E-13 | 7.8E-11 |
| GO:0031224 | intrinsic component of membrane | 2.72E-13 | 1.03E-10 |
| GO:0044425 | membrane part | 1.01E-9 | 2.56E-7 |

**Supplementary file 5.** Gene ontology (GO) enrichment analysis of plasma proteins with low inter-individual variability. The proteins were ranked from smallest to largest inter-individual variation based on their coefficient of variation. The top of the list (least varying) was enriched for proteins linked to humoral activation proteins and receptor activity.
