# Supplementary material for: In-depth human plasma proteome analysis captures tissue proteins and transfer of protein variants across the placenta
Source: eLife. 2019 Apr 8;8:e41608. doi: 10.7554/eLife.41608 (PMC6519984; doi:10.7554/eLife.41608)
Supplement: Supplementary file 6. — The proteins were ranked from highest to smallest inter-individual variation based on their coefficient of variation. The top of the list (most varying) was enriched for lipid transport proteins. [file elife-41608-supp6.docx]

| **Process** |  |  |  |
| --- | --- | --- | --- |
| **GO term** | **Description** | **P-value** | **FDR q-value** |
| GO:0006869 | lipid transport | 7.58E-8 | 5.46E-4 |
| GO:0006996 | organelle organization | 7.86E-8 | 2.83E-4 |
| GO:0034370 | triglyceride-rich lipoprotein particle remodeling | 1.31E-7 | 3.14E-4 |
|  |  |  |  |
| **Function** |  |  |  |
| **GO term** | **Description** | **P-value** | **FDR q-value** |
| GO:0008092 | cytoskeletal protein binding | 5.88E-10 | 8.3E-7 |
| GO:0005543 | phospholipid binding | 4.6E-7 | 3.25E-4 |
| GO:0001882 | nucleoside binding | 1.09E-6 | 5.12E-4 |
|  |  |  |  |
| **Component** |  |  |  |
| **GO term** | **Description** | **P-value** | **FDR q-value** |
| GO:0005829 | cytosol | 4.76E-20 | 3.6E-17 |
| GO:0005856 | cytoskeleton | 1.73E-12 | 6.55E-10 |
| GO:0044430 | cytoskeletal part | 6.18E-10 | 1.56E-7 |

**Supplementary file 6**. Gene ontology (GO) enrichment analysis of plasma proteins with high inter-individual variability. The proteins were ranked from highest to smallest inter-individual variation based on their coefficient of variation. The top of the list (most varying) was enriched for lipid transport proteins.
