# Supplementary material for: In-depth human plasma proteome analysis captures tissue proteins and transfer of protein variants across the placenta
Source: eLife. 2019 Apr 8;8:e41608. doi: 10.7554/eLife.41608 (PMC6519984; doi:10.7554/eLife.41608)
Supplement: Supplementary file 8. — Plasma from the same individual ‘not pregnant’: six entries, plasma ‘third trimester’: 30 entries (four entries overlapping between not pregnant and third trimester), in total HPA db placenta enriched: 83 entries). [file elife-41608-supp8.docx]

| **Source** | **Protein class** | **# Genes in database*** | **# Hits, HiRIEF data (Symbolcentric)** | **Expected #false hits, 1% FDR** |
| --- | --- | --- | --- | --- |
| proteinatlas.org | FDA approved drug targets | 646 | 143 |  |
|  | Cancer-related genes | 555 | 73 |  |
|  | Tissue enriched proteins | 2489 | 304 |  |
|  | *adipose tissue* | 26 | 6 | 0,26 |
|  | *adrenal gland* | 38 | 6 | 0,38 |
|  | *appendix* | 2 | 0 | 0,02 |
|  | *bone marrow* | 84 | 15 | 0,84 |
|  | *breast* | 23 | 23 | 0,23 |
|  | *cerebral cortex* | 381 | 44 | 3,81 |
|  | *duodenum* | 6 | 2 | 0,06 |
|  | *endometrium* | 4 | 0 | 0,04 |
|  | *esophagus* | 48 | 15 | 0,48 |
|  | *fallopian tube* | 66 | 2 | 0,66 |
|  | *gallbladder* | 7 | 1 | 0,07 |
|  | *heart muscle* | 33 | 3 | 0,33 |
|  | *kidney* | 70 | 4 | 0,7 |
|  | *liver* | 170 | 92 | 1,7 |
|  | *lung* | 20 | 4 | 0,2 |
|  | *ovary* | 7 | 1 | 0,07 |
|  | *pancreas* | 37 | 13 | 0,37 |
|  | *placenta* | 83 | 24 | 0,83 |
|  | *prostate* | 21 | 2 | 0,21 |
|  | *rectum* | 1 | 0 | 0,01 |
|  | *salivary gland* | 40 | 7 | 0,4 |
|  | *skeletal muscle* | 106 | 22 | 1,06 |
|  | *skin* | 95 | 22 | 0,95 |
|  | *small intestine* | 6 | 1 | 0,06 |
|  | *smooth muscle* | 1 | 0 | 0,01 |
|  | *spleen* | 8 | 1 | 0,08 |
|  | *stomach* | 31 | 4 | 0,31 |
|  | *testis* | 1057 | 10 | 10,57 |
|  | *thyroid gland* | 28 | 3 | 0,28 |
|  | *tonsil* | 7 | 0 | 0,07 |
|  | *urinary bladder* | 6 | 0 | 0,06 |
|  | Cytokines (AND pred secreted) | 36 | 4 |  |
|  | Kinases | 501 | 73 |  |
|  | RNA editing | 14 | 1 |  |
|  | Transcription factors | 1510 | 8 |  |
| Anderson 2002 | Classical plasma proteins | 33 | 28 (IgM, A, G not included) |  |
| Anderson 2002 | Tissue leakage proteins | 18 | 9 |  |

**Supplementary file 8.** Summary of protein classes used for the analyses in **Fig. 3b-c and 4a-d**. Plasma from the same individual “not pregnant”: 6 entries, plasma “third trimester”: 30 entries (4 entries overlapping between not pregnant and 3^rd^ trimester), in total HPA db placenta enriched: 83 entries).
