# Supplementary material for: In-depth human plasma proteome analysis captures tissue proteins and transfer of protein variants across the placenta
Source: eLife. 2019 Apr 8;8:e41608. doi: 10.7554/eLife.41608 (PMC6519984; doi:10.7554/eLife.41608)
Supplement: Supplementary file 10. [file elife-41608-supp10.docx]

| **Gene** | **Direction of transfer** | **Mother/Baby pair** | **Size (Da)** | **pI** | **Gravy score** |
| --- | --- | --- | --- | --- | --- |
| AGT | m>b | m>b_SO | 53154 | 5.87 | 0.064536082474227 |
| APOB | m<>b | b>m_ST | 515605 | 6.58 | -0.23145454545455 |
| APOB | m<>b | m>b_SO | 515605 | 6.58 | -0.23145454545455 |
| APOH | b>m | b>m_SO | 38298 | 8.34 | -0.2736231884058 |
| C14orf37 | b>m | b>m_ST | 84173 | 4.26 | -0.53049095607235 |
| C1RL | m>b | m>b_SO | 53498 | 6.75 | -0.36447638603696 |
| C3 | b>m | b>m_SO | 187148 | 6.02 | -0.21454545454545 |
| C4BPA | m>b | m>b_SO | 67033 | 7.15 | -0.47671691792295 |
| C7 | b>m | b>m_ST | 93518 | 6.09 | -0.4864768683274 |
| CLEC3B | m>b | m>b_ST | 22537 | 5.52 | -0.29851485148515 |
| FGFBP2 | m>b | m>b_SO | 24581 | 9.15 | -0.6762331838565 |
| GAA | b>m | b>m_SO | 105324 | 5.62 | -0.12920168067227 |
| GAA | b>m | b>m_ST | 105324 | 5.62 | -0.12920168067227 |
| GAA | b>m | b>m_ST | 105324 | 5.62 | -0.12920168067227 |
| HRG | b>m | b>m_SO | 59578 | 7.09 | -0.9567619047619 |
| HSPG2 | m>b | m>b_SO | 468830 | 6.06 | -0.41984615384615 |
| HSPG2 | m>b | m>b_ST | 468830 | 6.06 | -0.41984615384615 |
| KRT2 | b>m | b>m_SO | 65433 | 8.07 | -0.47460815047022 |
| LNPEP | b>m | b>m_SO | 117349 | 5.5 | -0.19365853658537 |
| LRG1 | b>m | b>m_SO | 38178 | 6.45 | -0.14380403458213 |
| LRG1 | b>m | b>m_ST | 38178 | 6.45 | -0.14380403458213 |
| LTF | b>m | b>m_ST | 78182 | 8.5 | -0.33704225352113 |
| PIGR | m>b | m>b_SO | 83284 | 5.59 | -0.34070680628272 |
| PTPRG | b>m | b>m_ST | 162003 | 5.99 | -0.56463636363636 |
| RELN | m>b | m>b_ST | 388388 | 5.54 | -0.21209090909091 |
| SERPINB11 | m<>b | b>m_ST | 44099 | 8.42 | -0.20255102040816 |
| SERPINB11 | m<>b | m>b_SO | 44099 | 8.42 | -0.20255102040816 |
| SERPINF1 | b>m | b>m_ST | 46312 | 5.97 | -0.16387559808612 |
| SERPINF1 | b>m | b>m_ST | 46312 | 5.97 | -0.16387559808612 |
| SERPING1 | m>b | m>b_ST | 55154 | 6.09 | -0.125 |
| SERPING1 | m>b | m>b_ST | 55154 | 6.09 | -0.125 |
| SVEP1 | b>m | b>m_ST | 390170 | 5.32 | -0.39954545454545 |

**Supplementary file 10.** Brief summary of transfer protein properties in terms of hydrophilicity/hydrophobicity (as evaluated by Gravy score), protein size or pI.
