# Supplementary material for: In-depth human plasma proteome analysis captures tissue proteins and transfer of protein variants across the placenta
Source: eLife. 2019 Apr 8;8:e41608. doi: 10.7554/eLife.41608 (PMC6519984; doi:10.7554/eLife.41608)
Supplement: Supplementary file 12. [file elife-41608-supp12.docx]

| **Sample** | **gradient length** | **plate position** |  | **Sample** | **gradient length** | **plate position** |
| --- | --- | --- | --- | --- | --- | --- |
| fraction_01 | 50 min | A9 |  | fraction_37 | 50 min | E6 |
| fraction_02 | 50 min | A8 |  | fraction_38 | 70 min | E5 |
| fraction_03 | 70 min | A7 |  | fraction_39 | 70 min | E4 |
| fraction_04 | 70 min | B9 |  | fraction_40 | 50 min | F6 |
| fraction_05 | 90 min | B8 |  | fraction_41 | 50 min | F5 |
| fraction_06 | 90 min | B7 |  | fraction_42 | 50 min | F4 |
| fraction_07 | 110 min | C9 |  | fraction_43 | accumulate in trap column | G6 |
| fraction_08 | 110 min | C8 |  | fraction_44 | accumulate in trap column | G5 |
| fraction_09 | 110 min | C7 |  | fraction_45 | accumulate in trap column | G4 |
| fraction_10 | 110 min | D9 |  | fraction_46 | accumulate in trap column | H6 |
| fraction_11 | 110 min | D8 |  | fraction_47 | accumulate in trap column | H5 |
| fraction_12 | 90 min | D7 |  | fraction_48 | accumulate in trap column | H4 |
| fraction_13 | 90 min | E9 |  | fraction_49 | accumulate in trap column | A3 |
| fraction_14 | 90 min | E8 |  | fraction_50 | 50 min | A2 |
| fraction_15 | 90 min | E7 |  | fraction_51 | 70 min | A1 |
| fraction_16 | 70 min | F9 |  | fraction_52 | 70 min | B3 |
| fraction_17 | 50 min | F8 |  | fraction_53 | accumulate in trap column | B2 |
| fraction_18 | 50 min | F7 |  | fraction_54 | accumulate in trap column | B1 |
| fraction_19 | 50 min | G9 |  | fraction_55 | accumulate in trap column | C3 |
| fraction_20 | accumulate in trap column | G8 |  | fraction_56 | accumulate in trap column | C2 |
| fraction_21 | accumulate in trap column | G7 |  | fraction_57 | accumulate in trap column | C1 |
| fraction_22 | accumulate in trap column | H9 |  | fraction_58 | accumulate in trap column | D3 |
| fraction_23 | accumulate in trap column | H8 |  | fraction_59 | accumulate in trap column | D2 |
| fraction_24 | accumulate in trap column | H7 |  | fraction_60 | accumulate in trap column | D1 |
| fraction_25 | accumulate in trap column | A6 |  | fraction_61 | accumulate in trap column | E3 |
| fraction_26 | accumulate in trap column | A5 |  | fraction_62 | accumulate in trap column | E2 |
| fraction_27 | 50 min | A4 |  | fraction_63 | accumulate in trap column | E1 |
| fraction_28 | 50 min | B6 |  | fraction_64 | 50 min | F3 |
| fraction_29 | 70 min | B5 |  | fraction_65 | 50 min | F2 |
| fraction_30 | 50 min | B4 |  | fraction_66 | 70 min | F1 |
| fraction_31 | 50 min | C6 |  | fraction_67 | accumulate in trap column | G3 |
| fraction_32 | accumulate in trap column | C5 |  | fraction_68 | accumulate in trap column | G2 |
| fraction_33 | accumulate in trap column | C4 |  | fraction_69 | accumulate in trap column | G1 |
| fraction_34 | accumulate in trap column | D6 |  | fraction_70 | accumulate in trap column | H3 |
| fraction_35 | accumulate in trap column | D5 |  | fraction_71 | 50 min | H2 |
| fraction_36 | 50 min | D4 |  | fraction_72 | 50 min | H1 |

**Supplementary file 12.** Pooling strategy for the condensed HiRIEF LC-MS/MS analysis used in the longitudinal female cohort.
