# Supplementary figures and images for: In-depth human plasma proteome analysis captures tissue proteins and transfer of protein variants across the placenta
Source: eLife. 2019 Apr 8;8:e41608. doi: 10.7554/eLife.41608 (PMC6519984; doi:10.7554/eLife.41608)

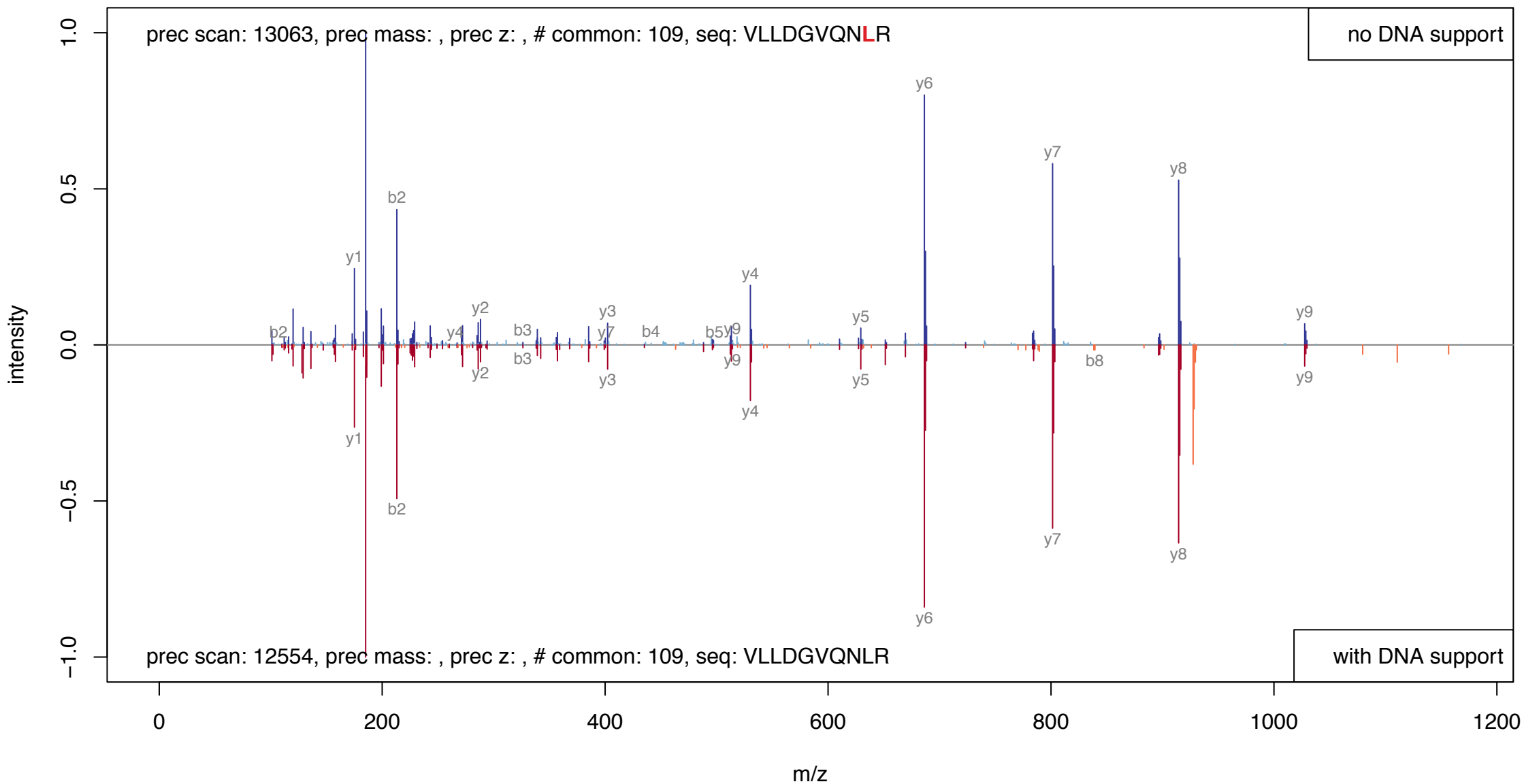

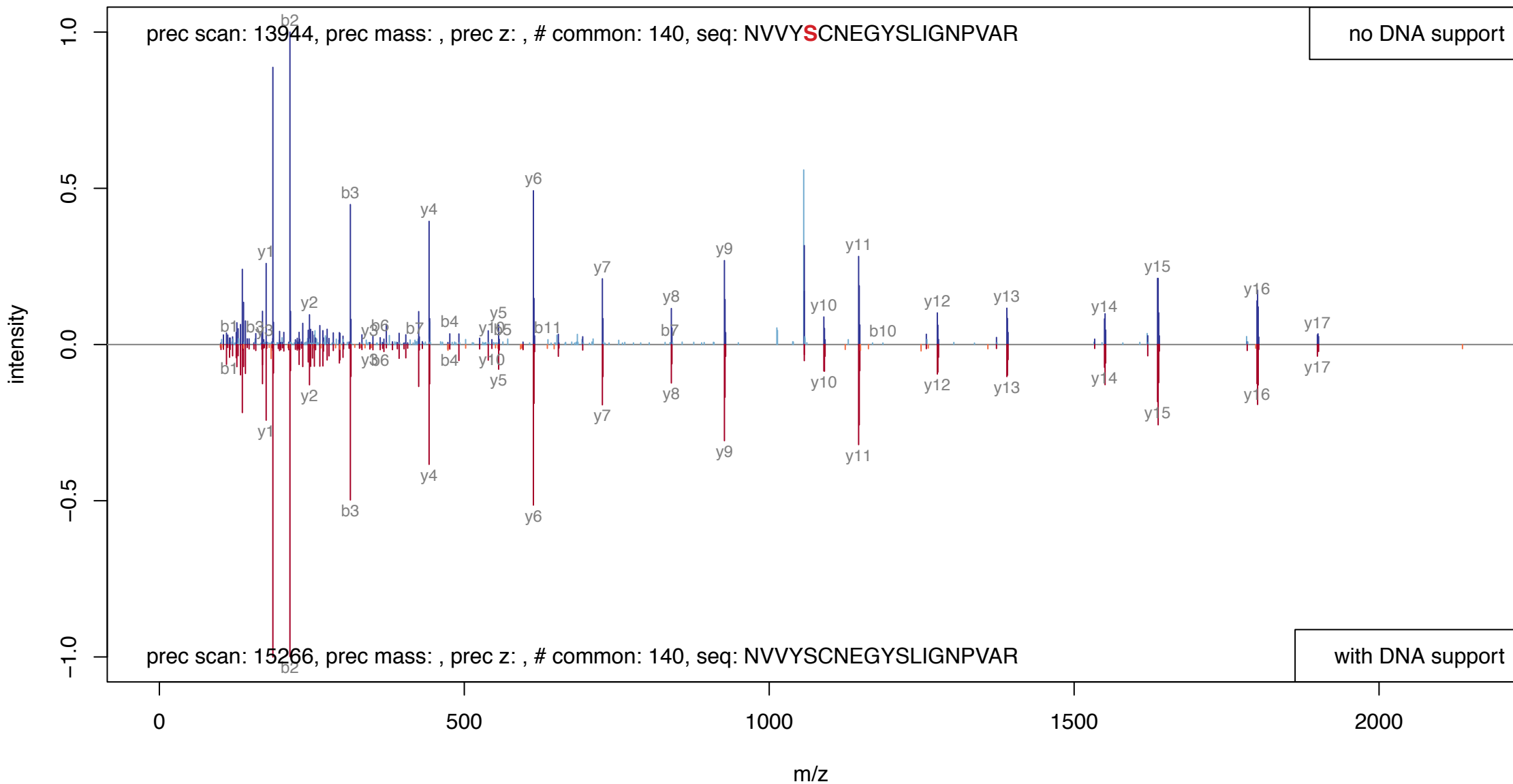

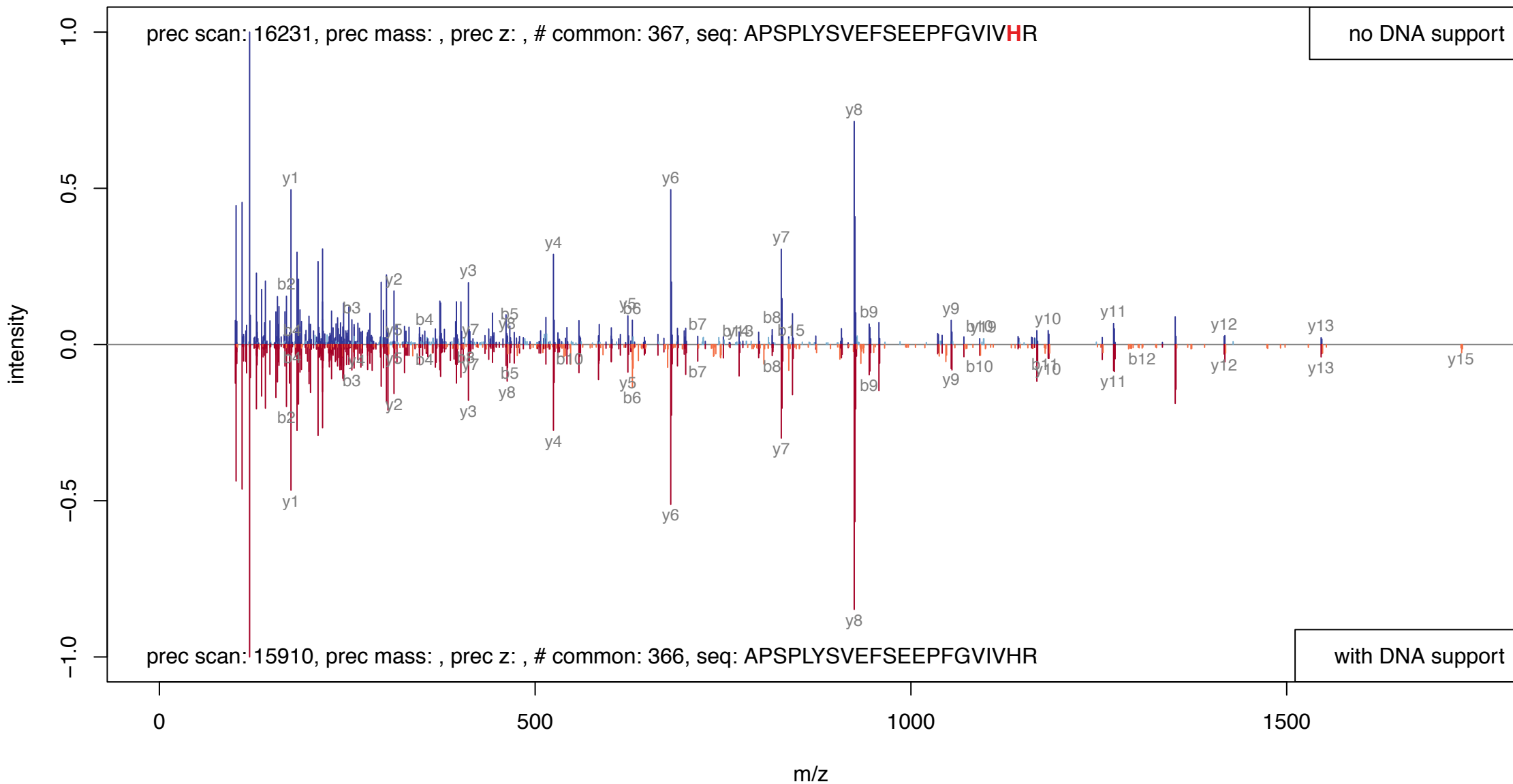

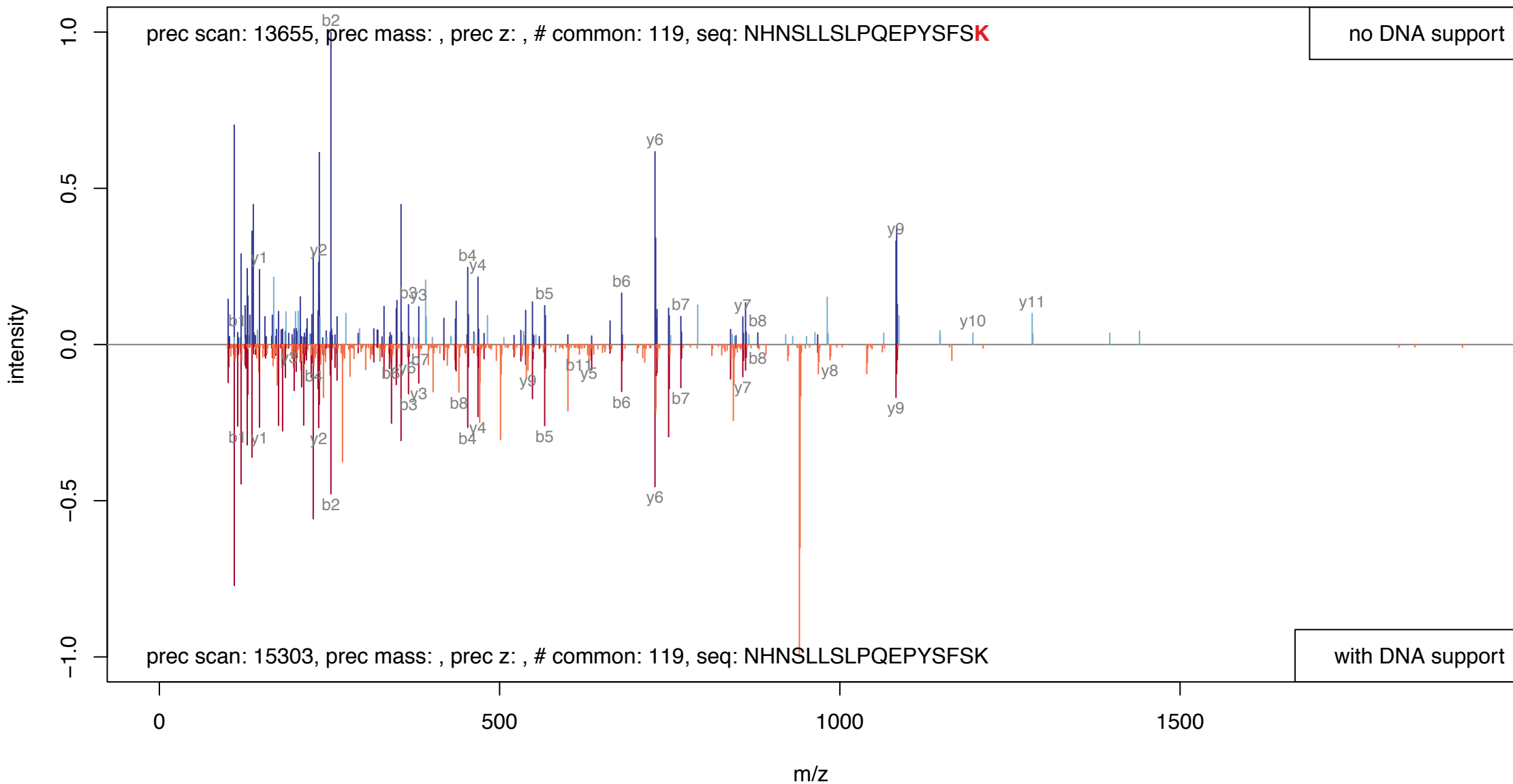

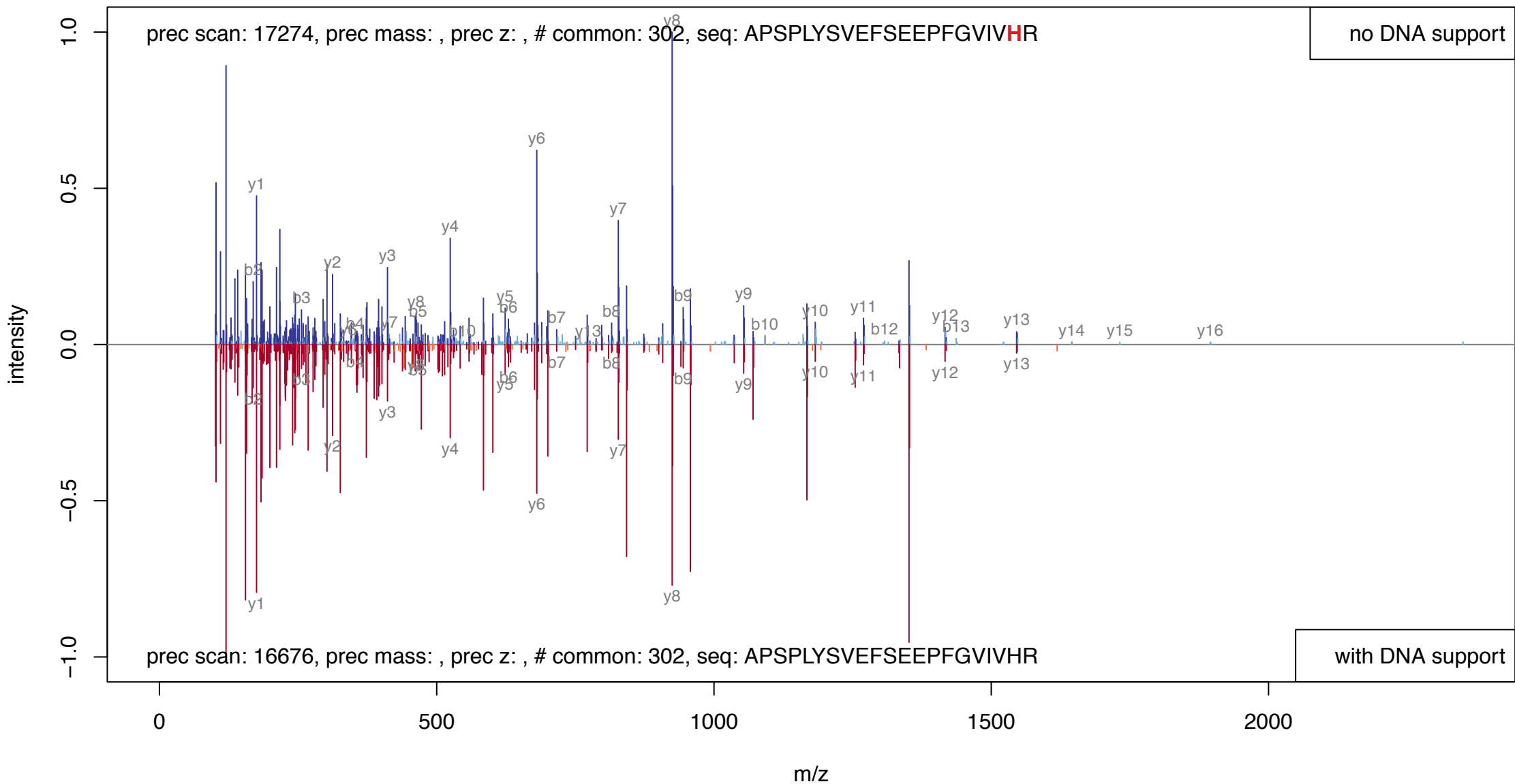

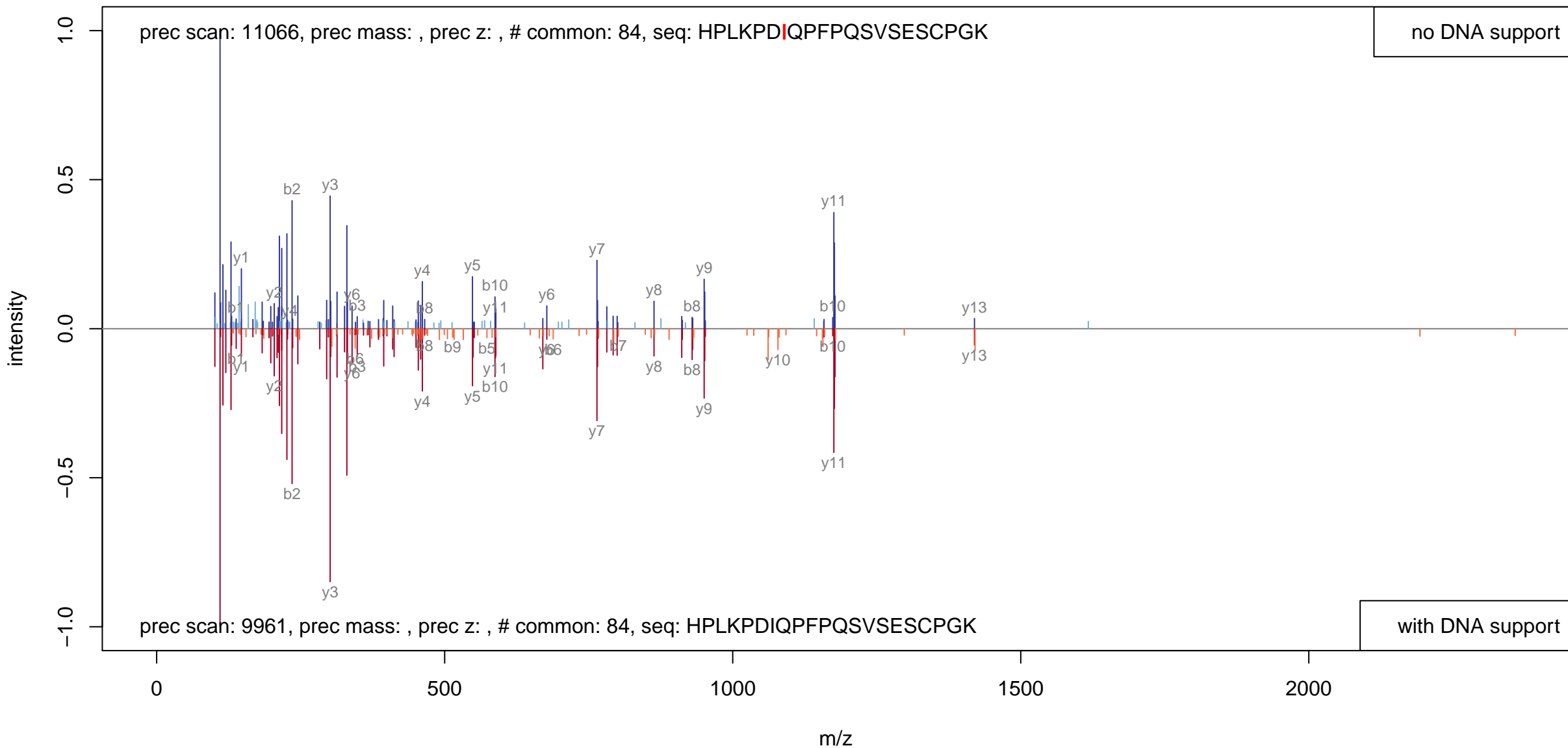

Incorrect match

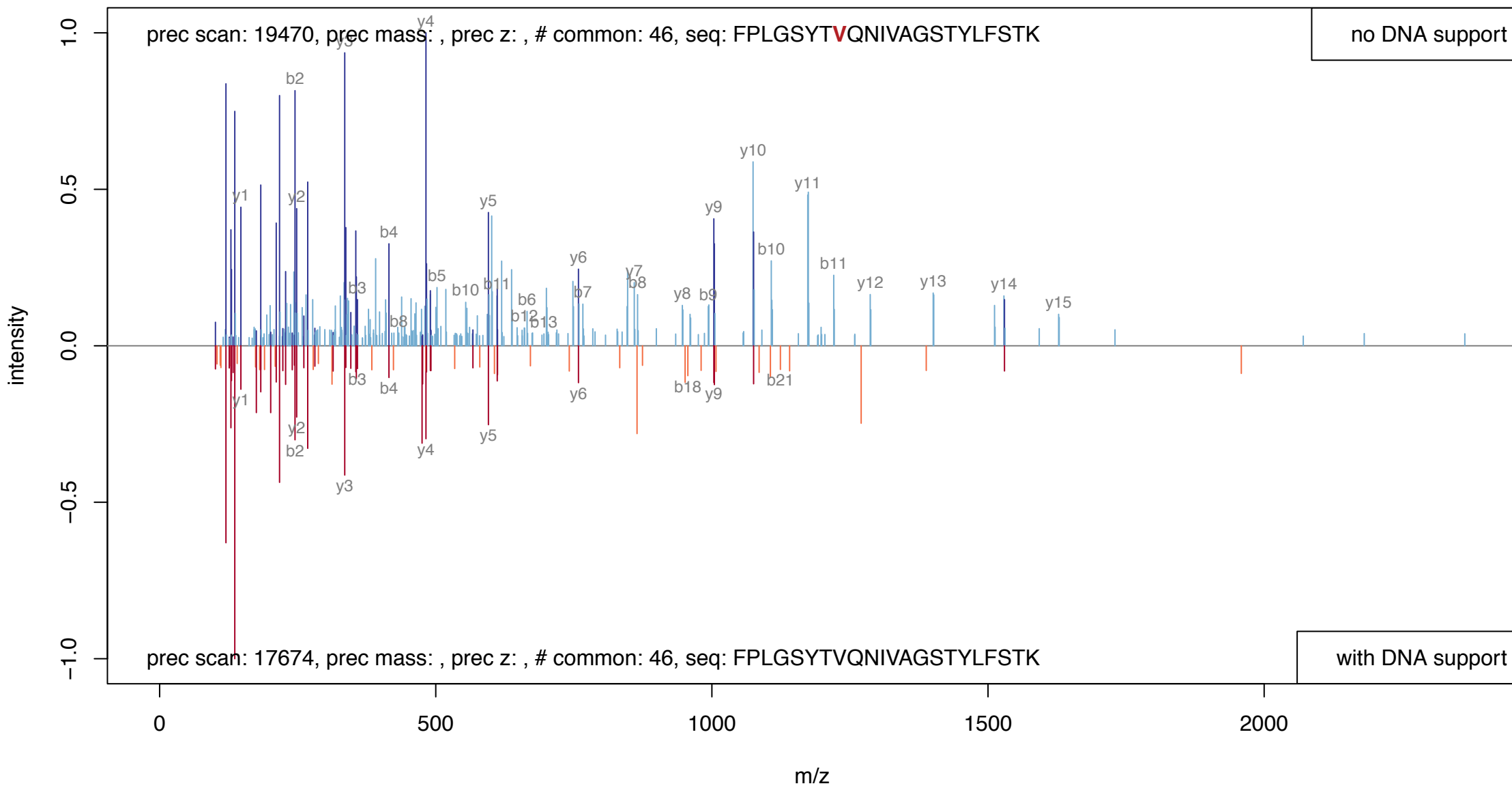

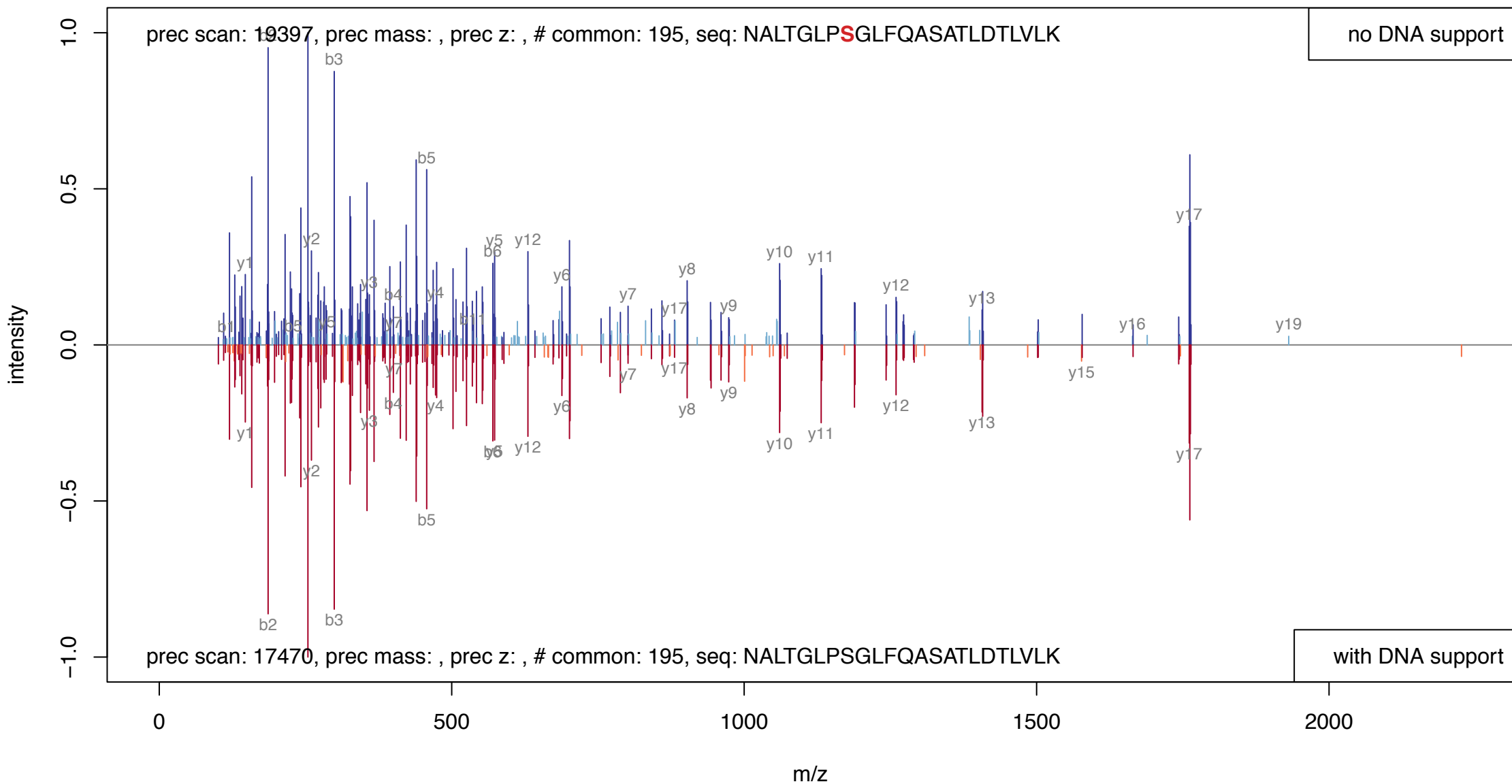

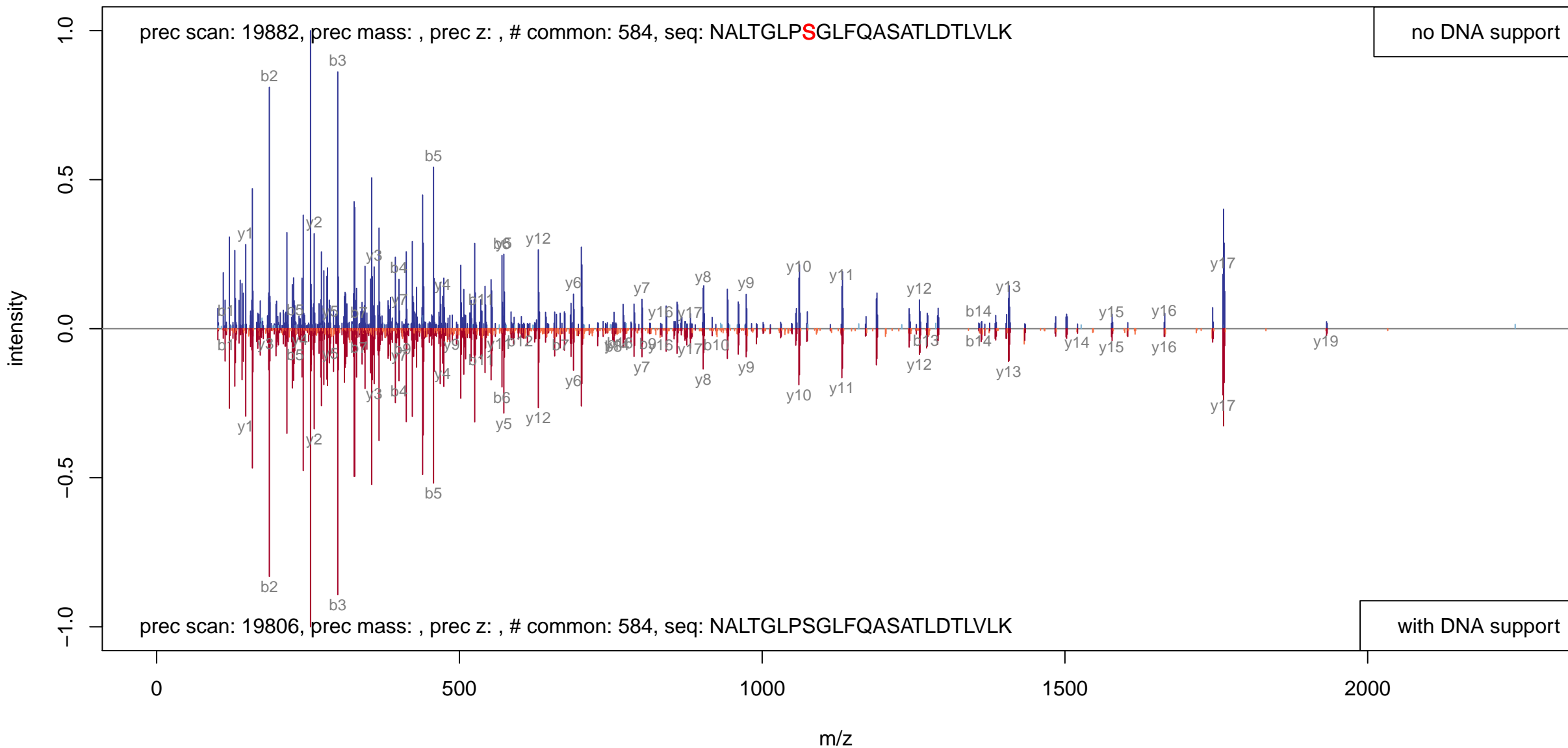

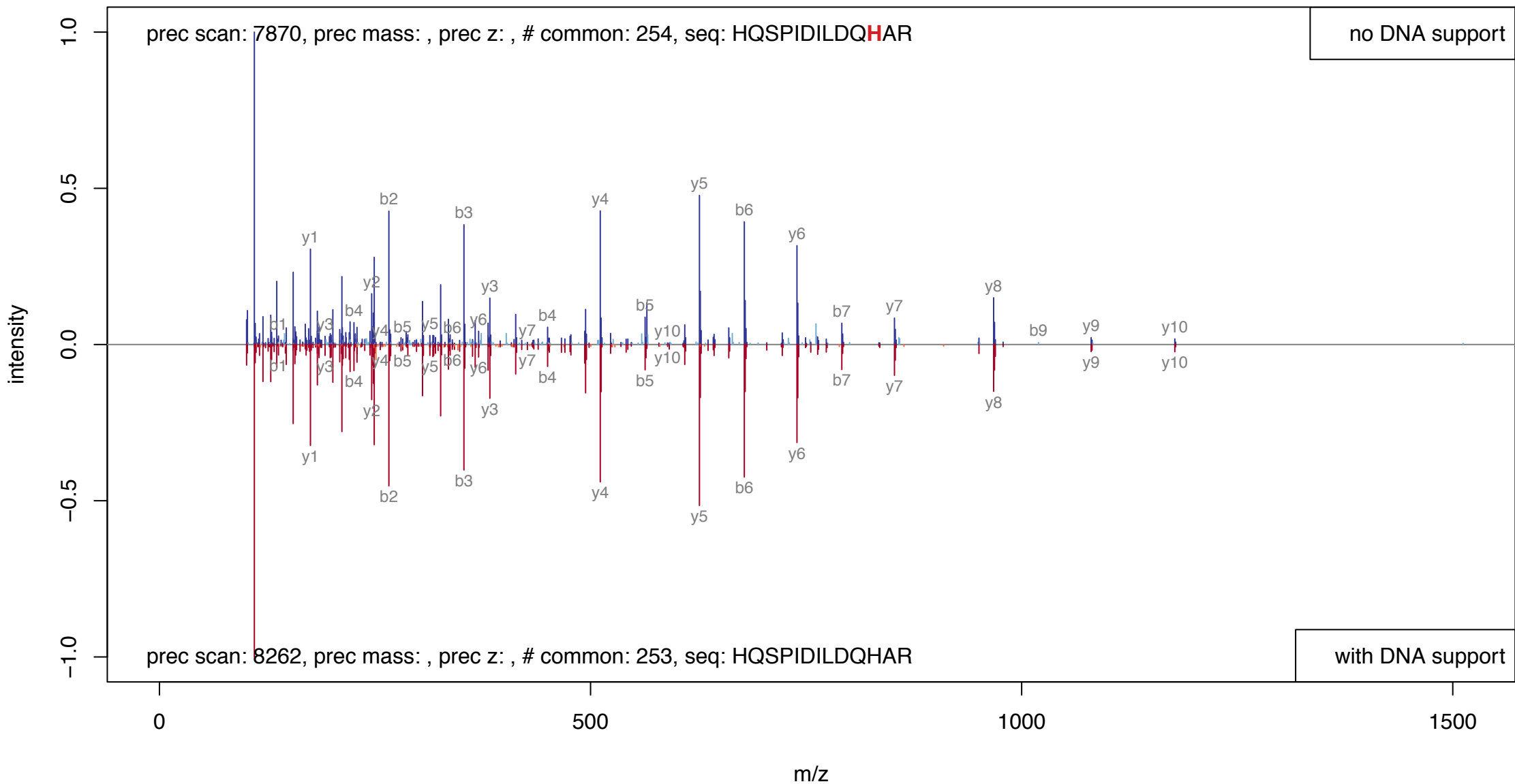

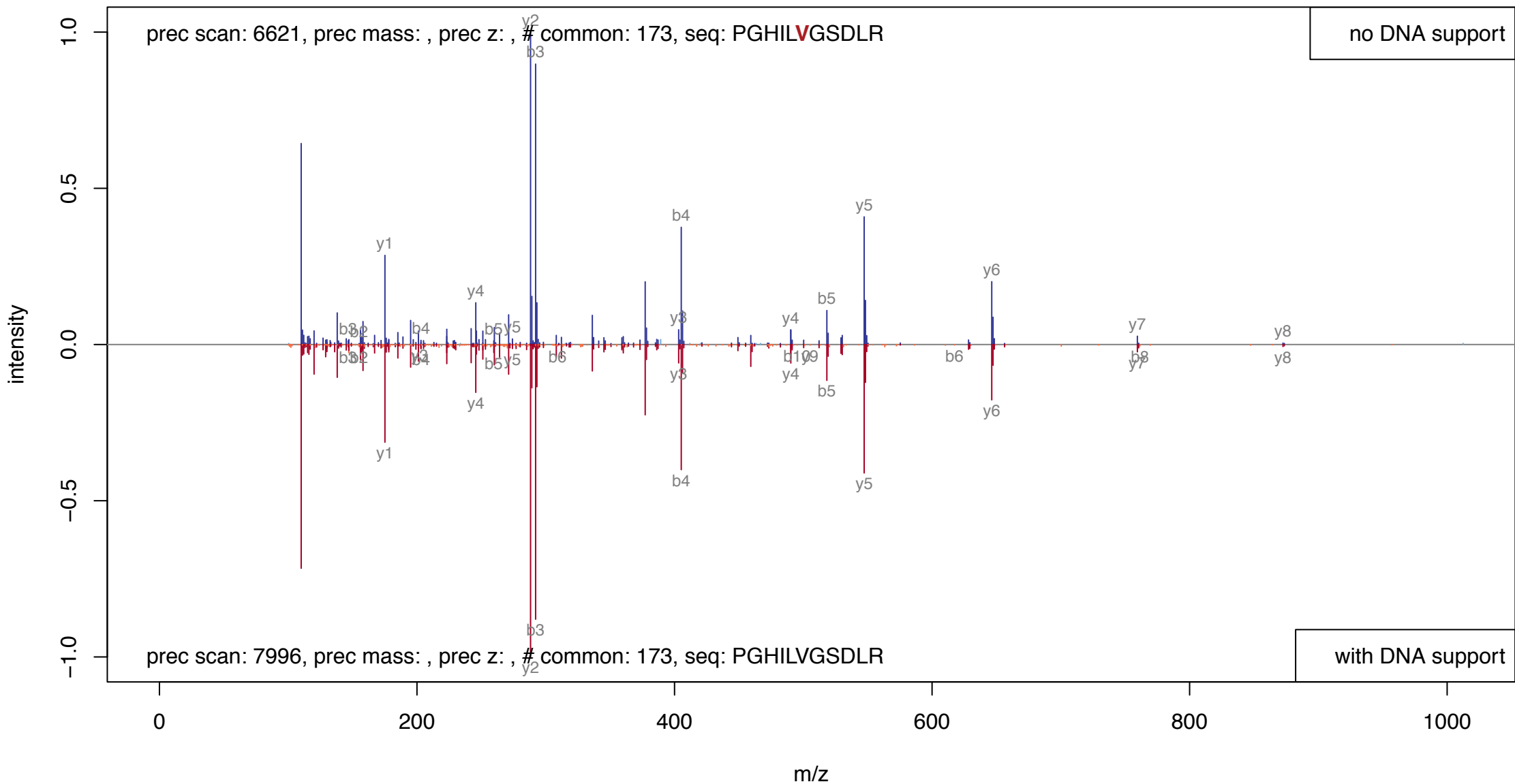

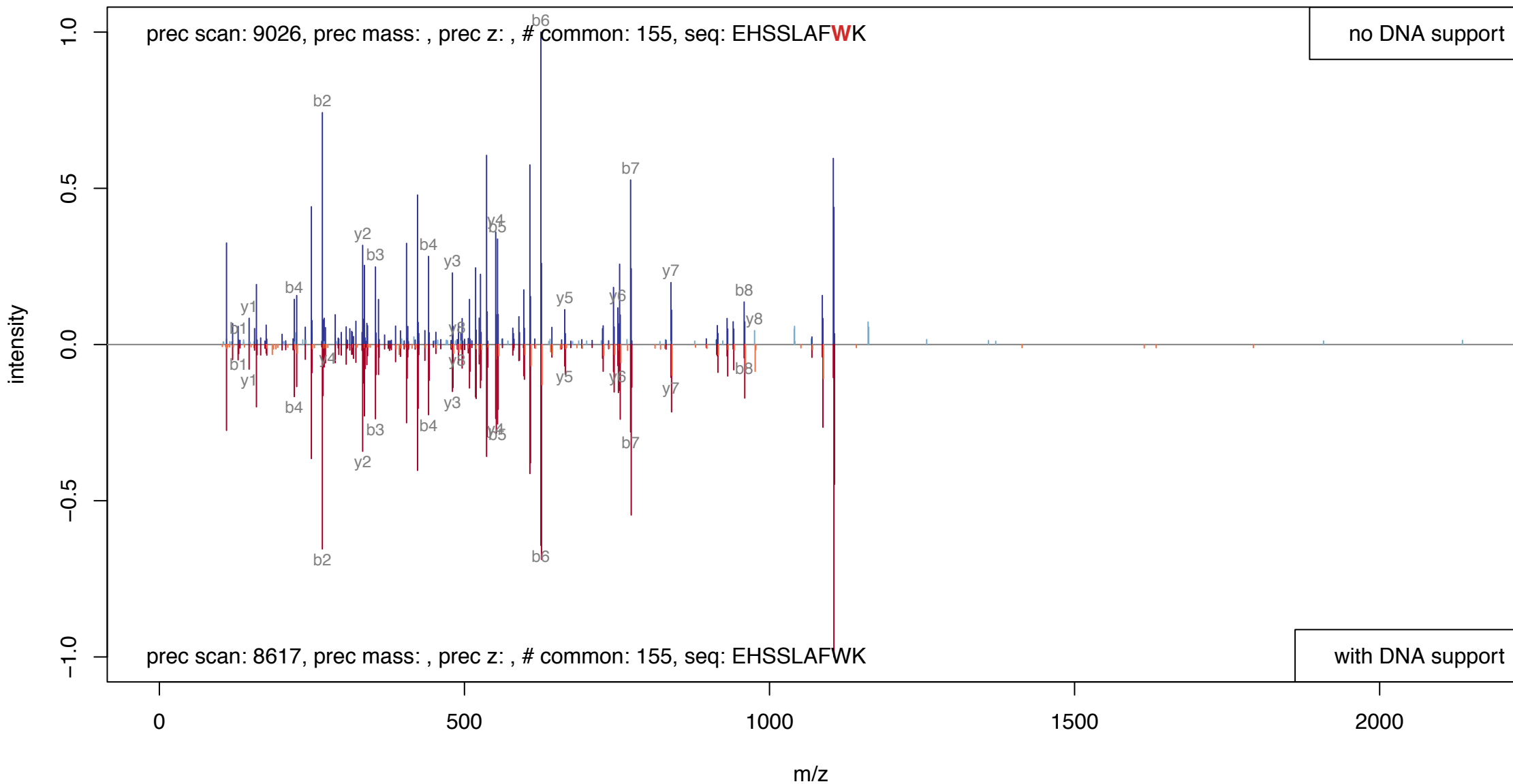

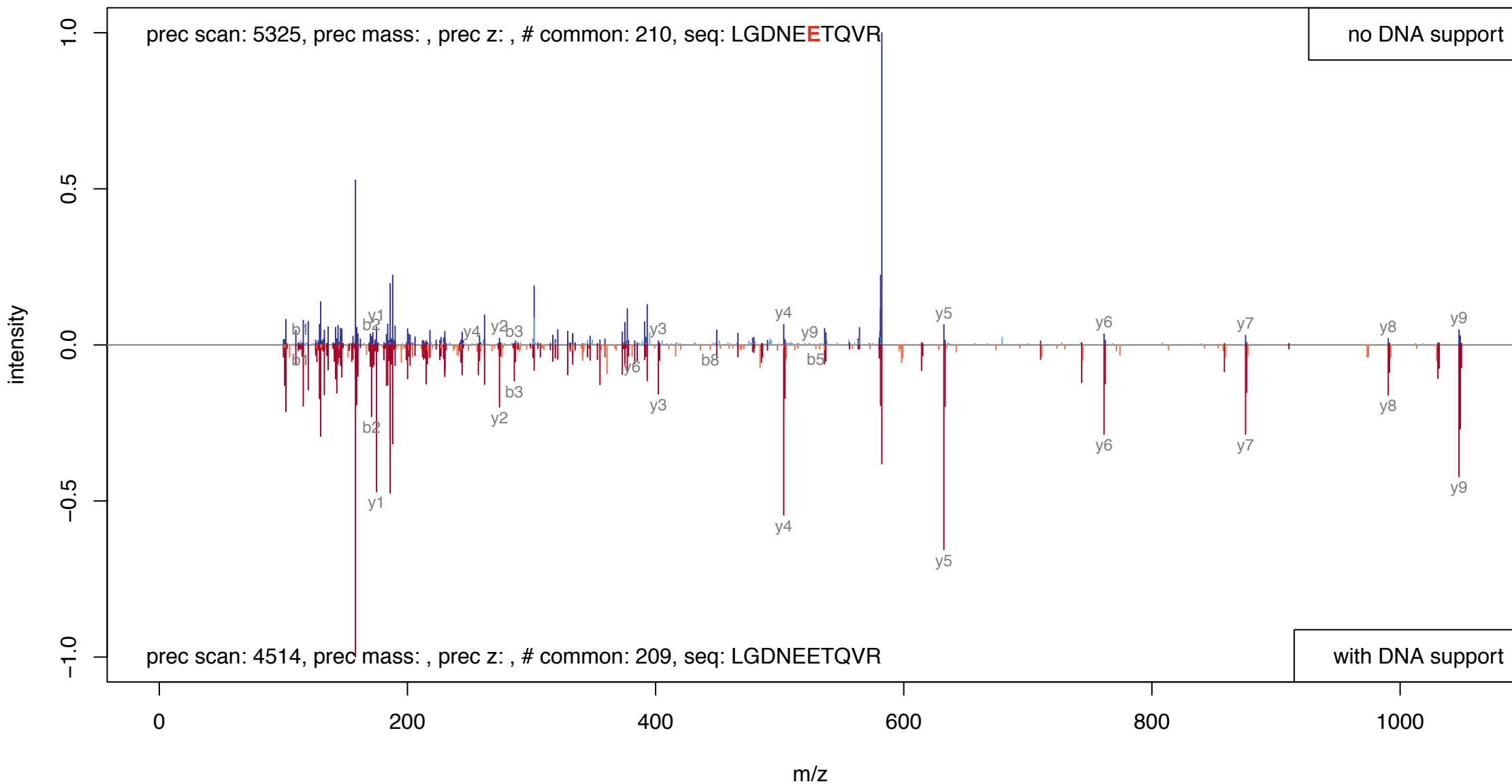

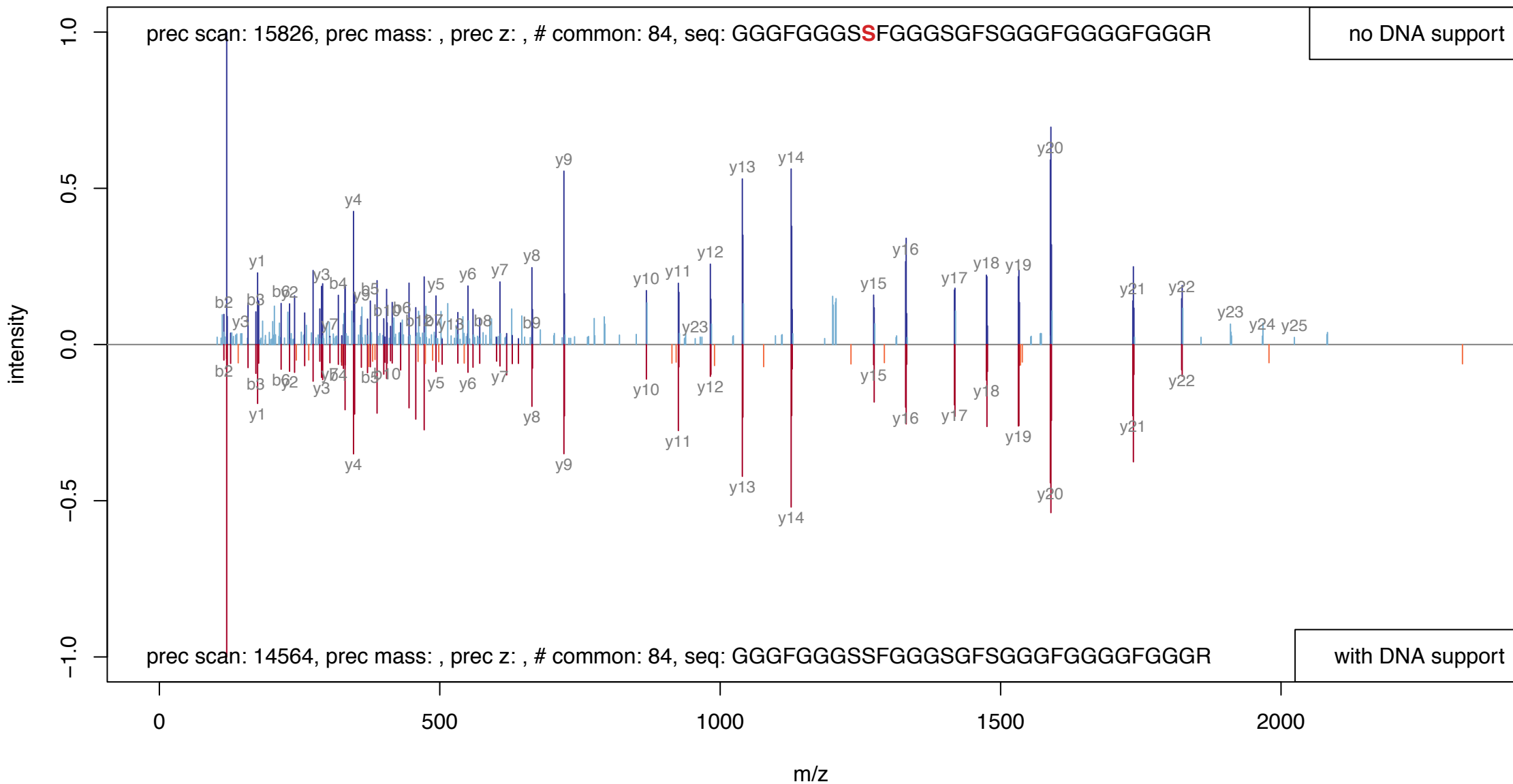

Incorrect match

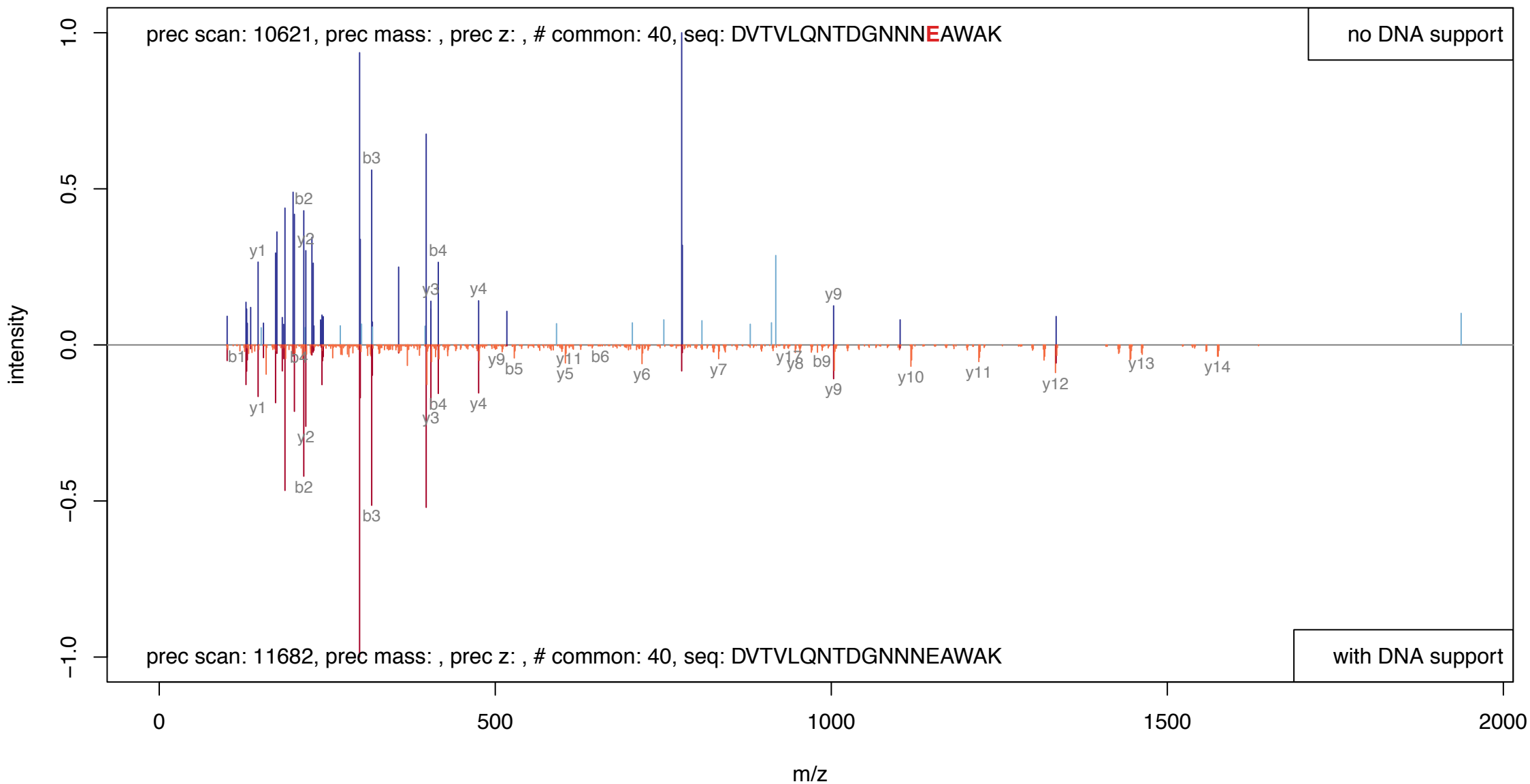

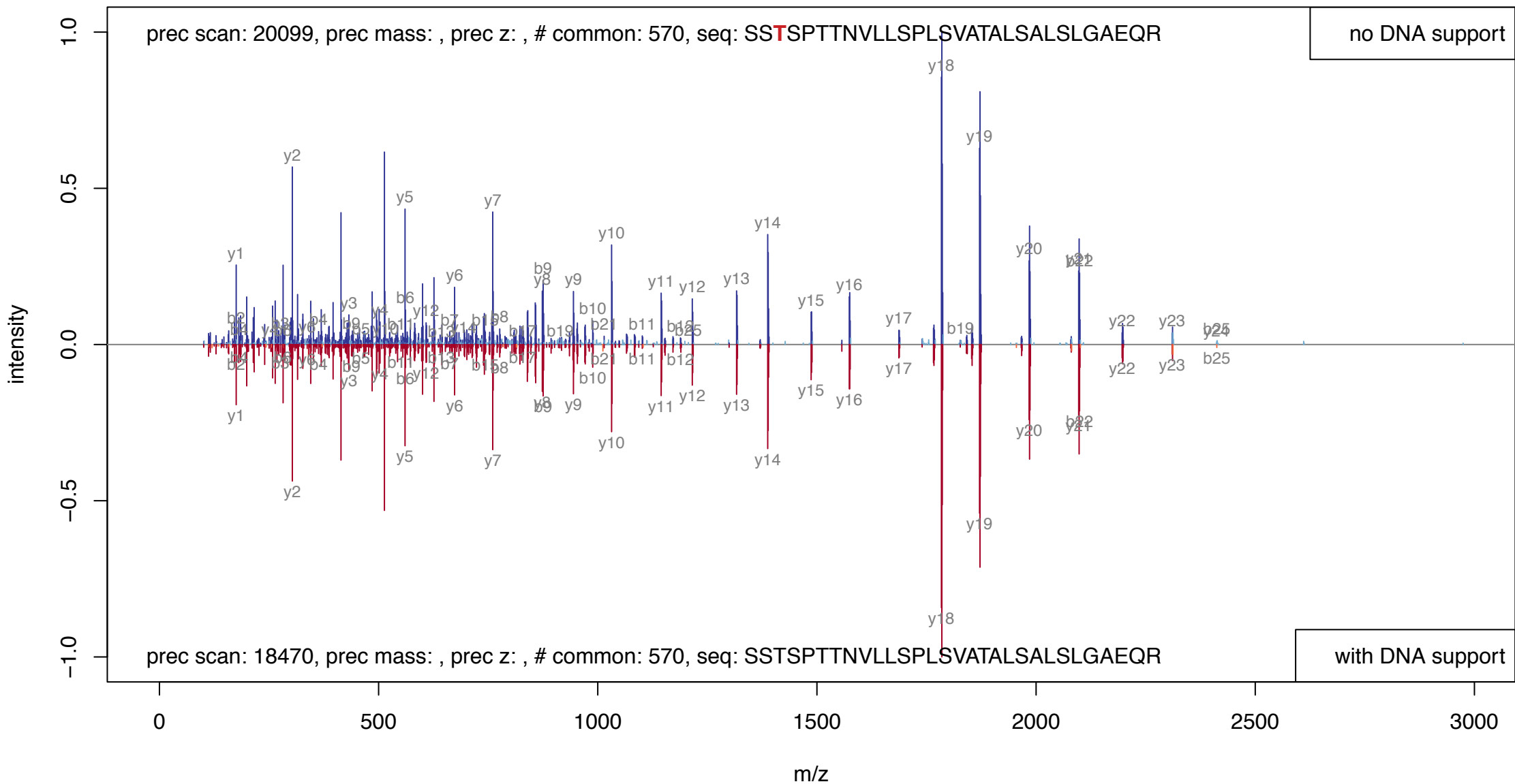

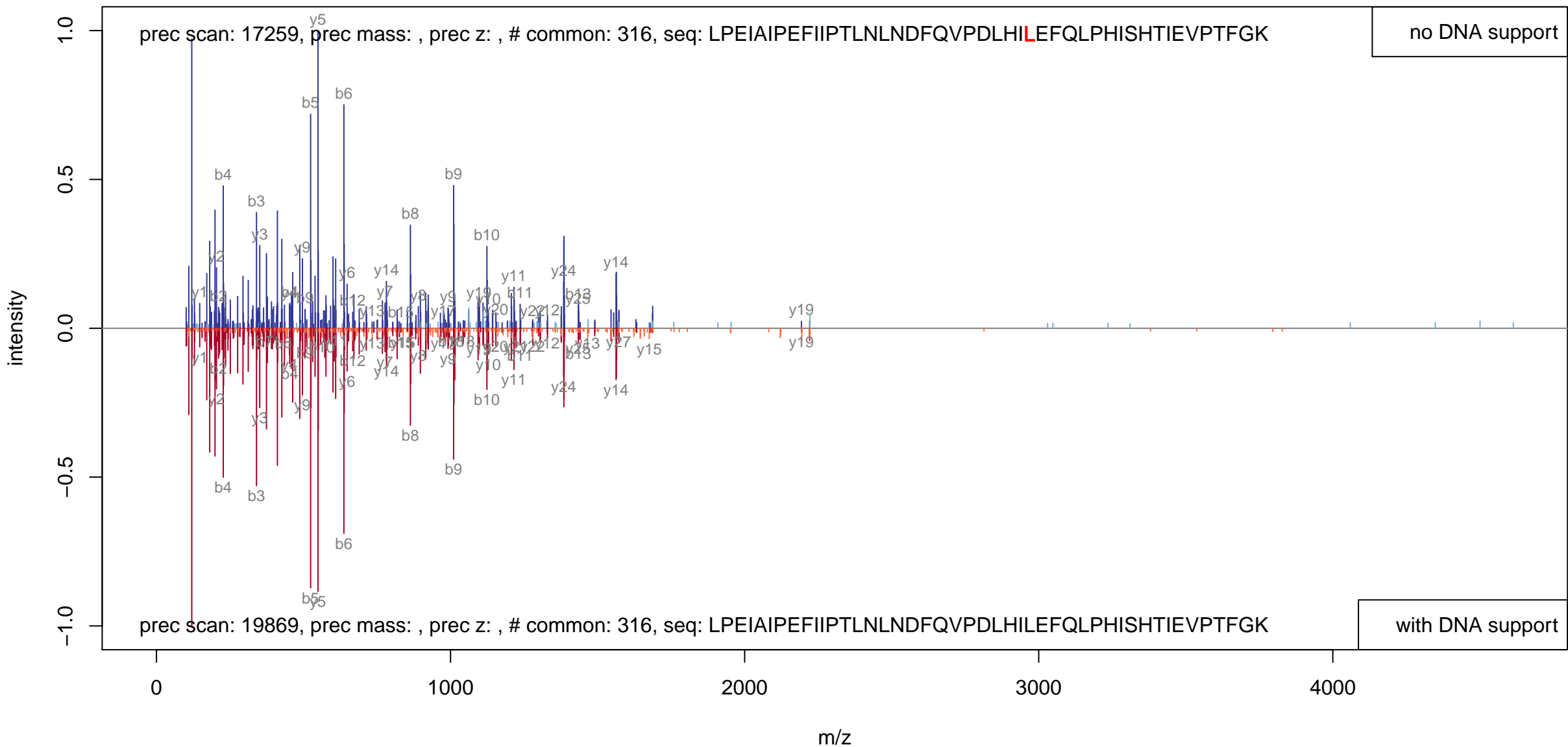

Incorrect match

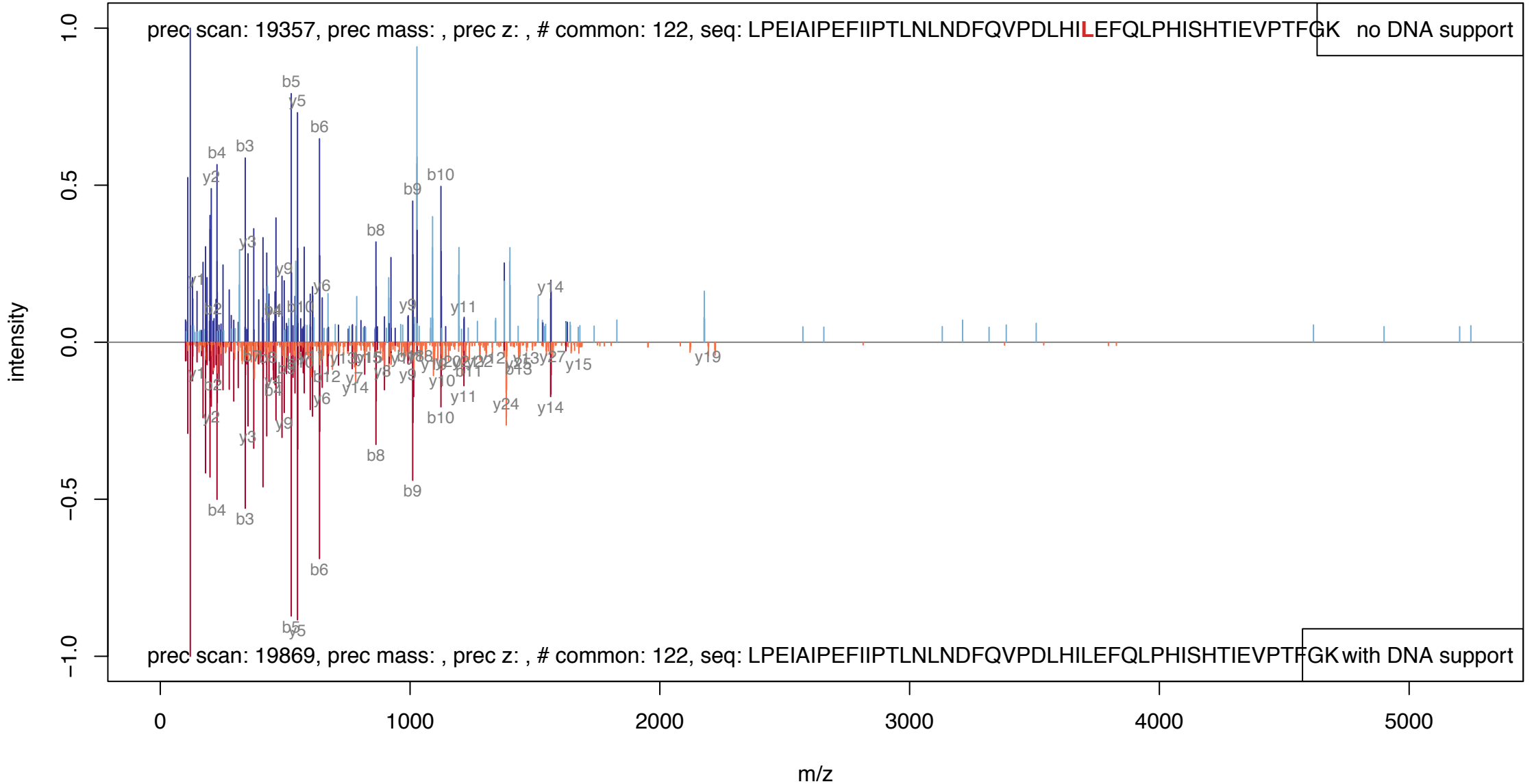

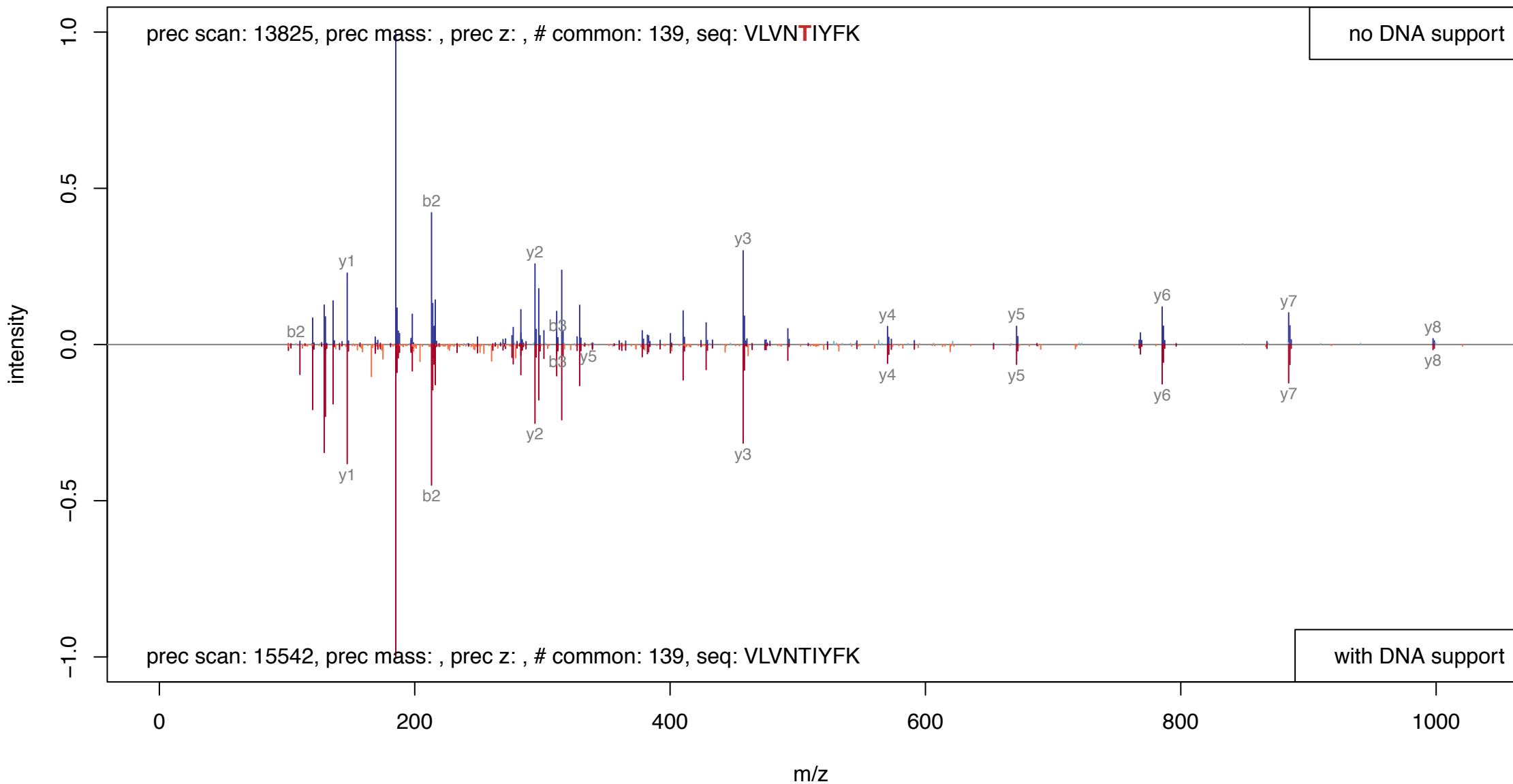

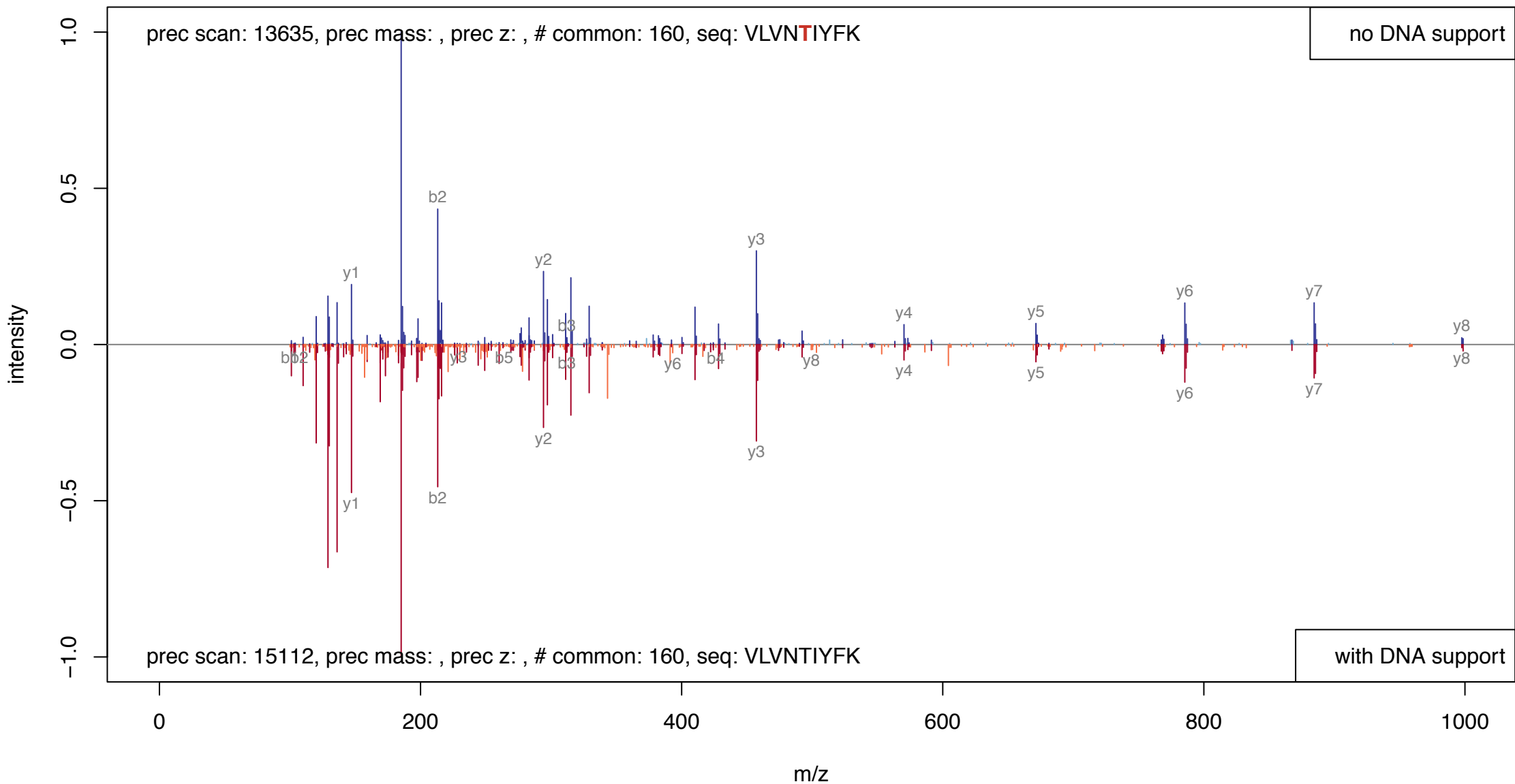

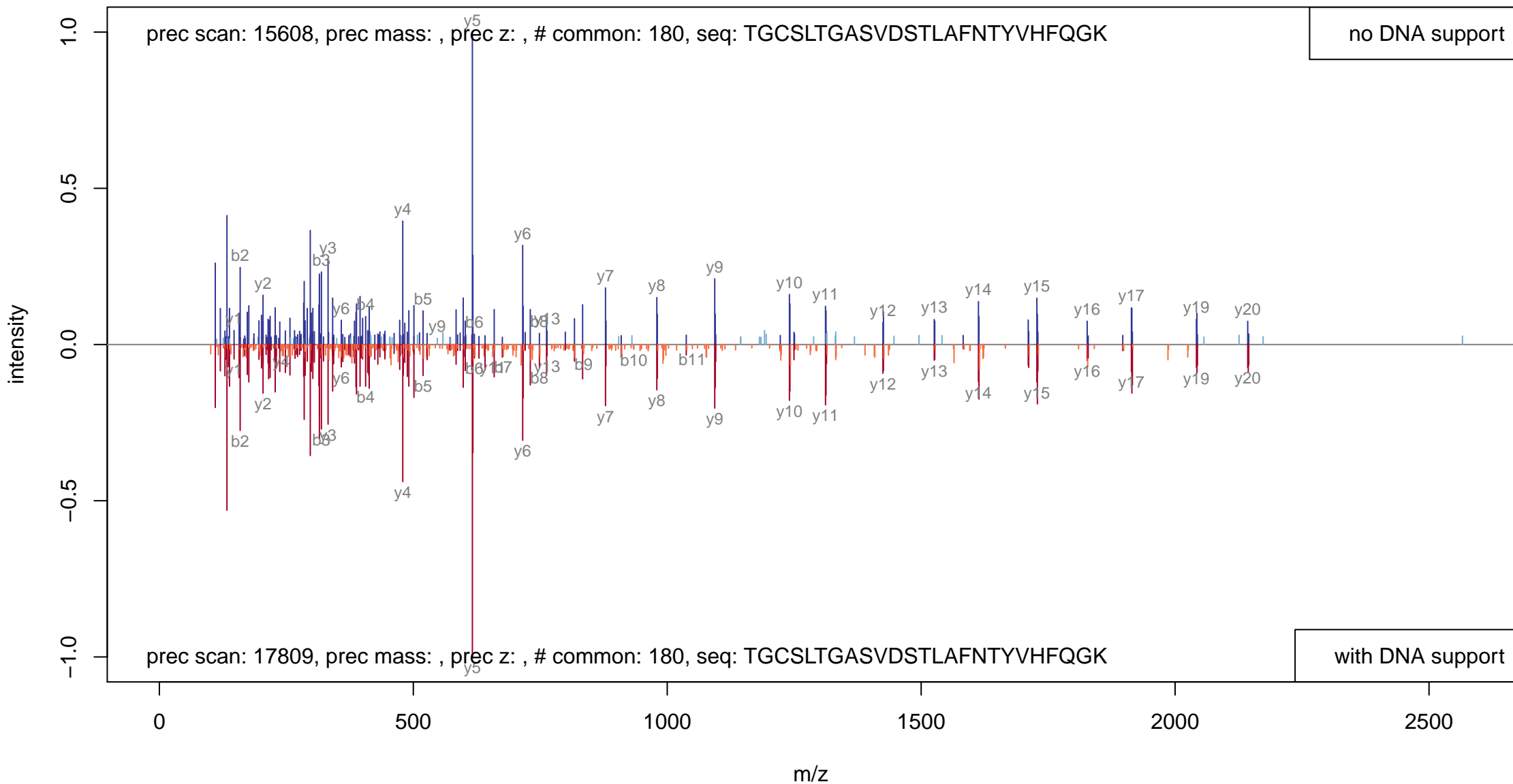

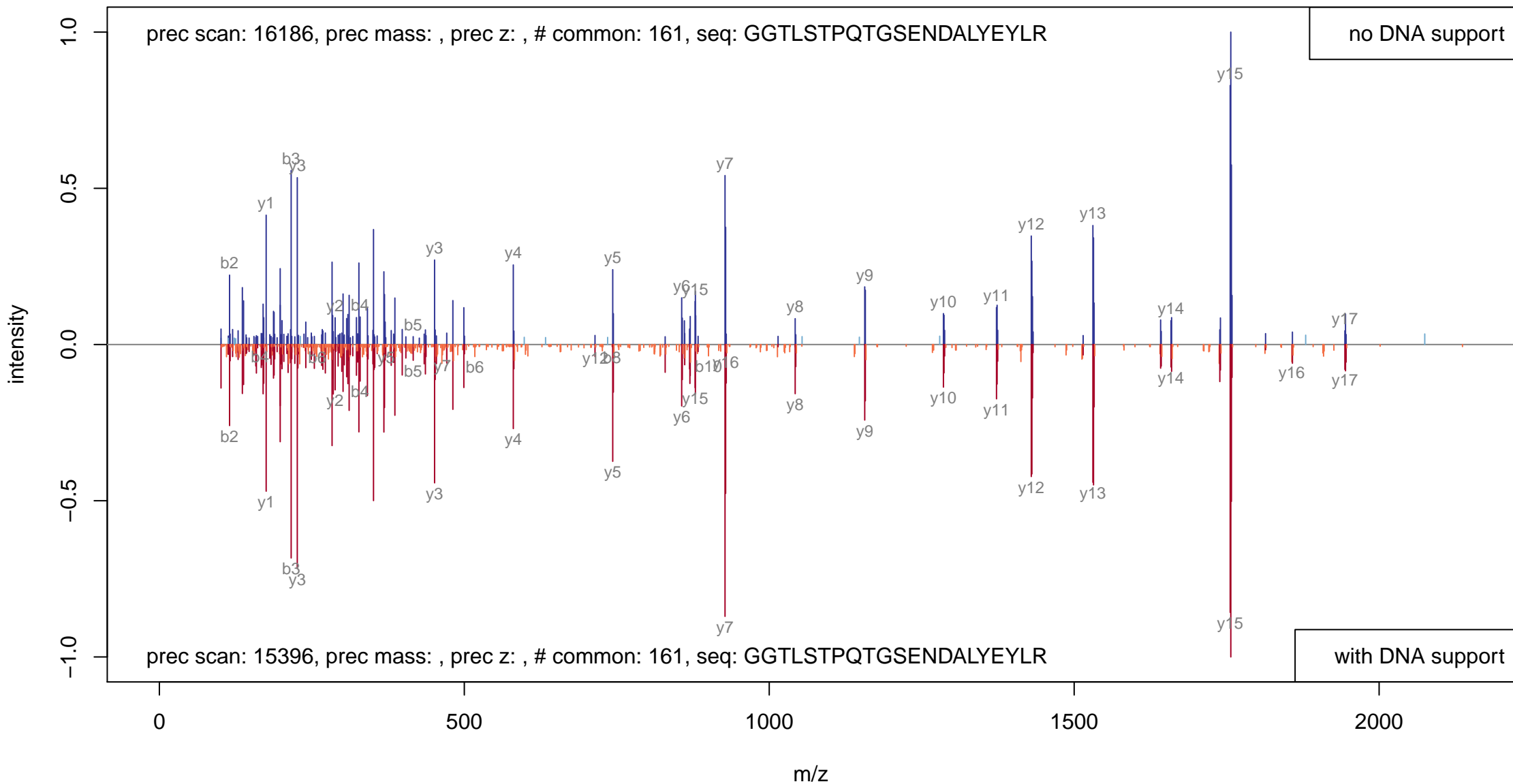

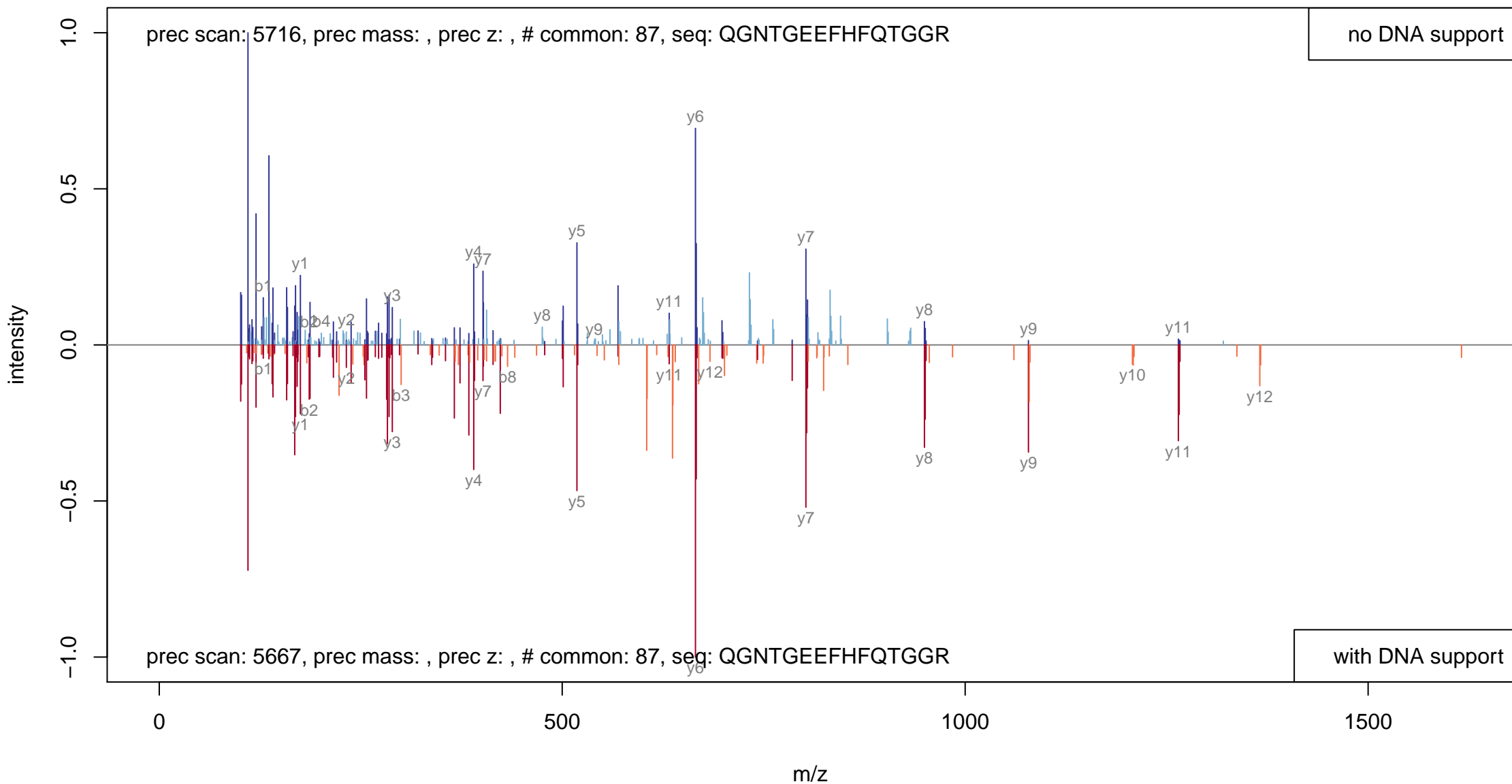

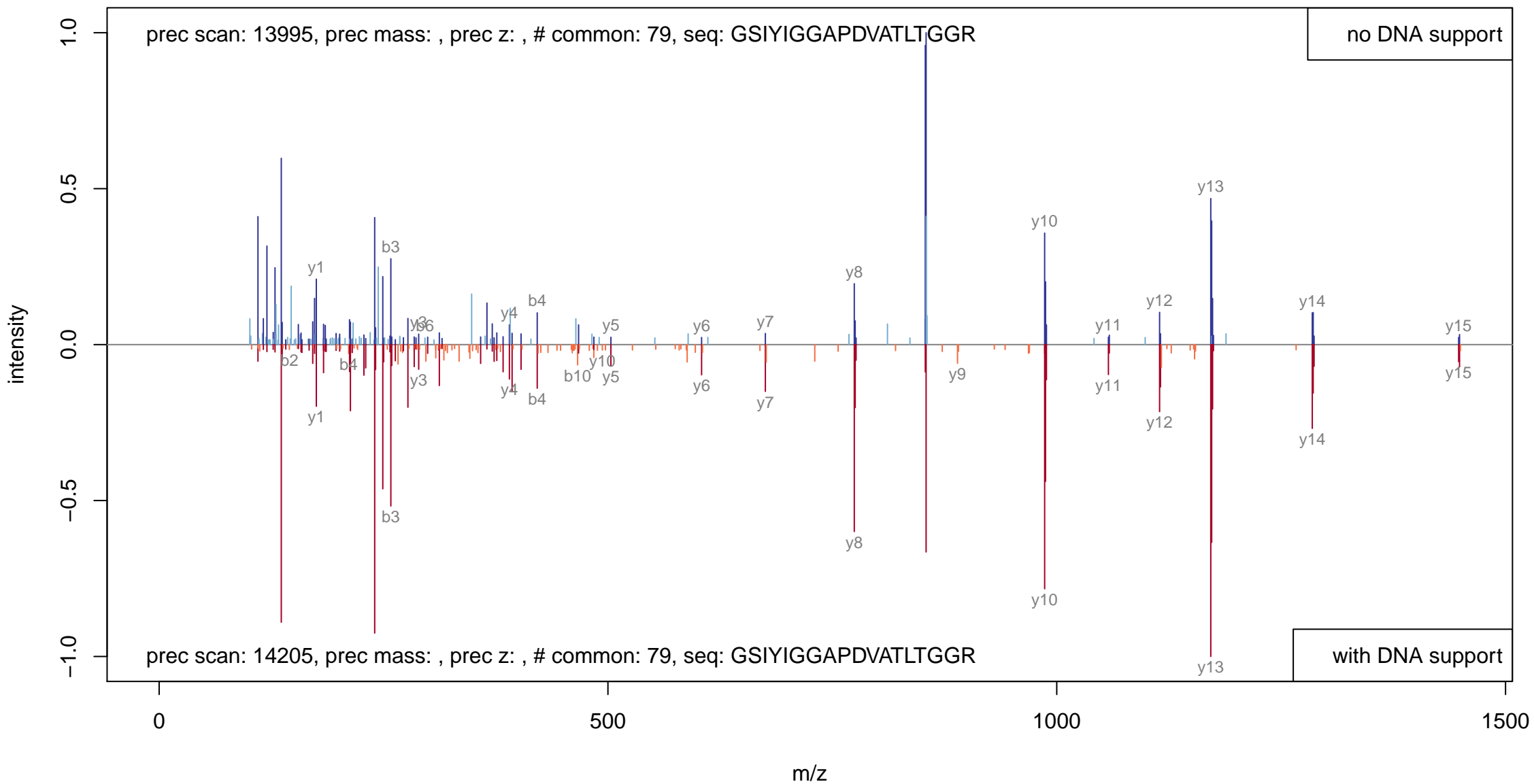

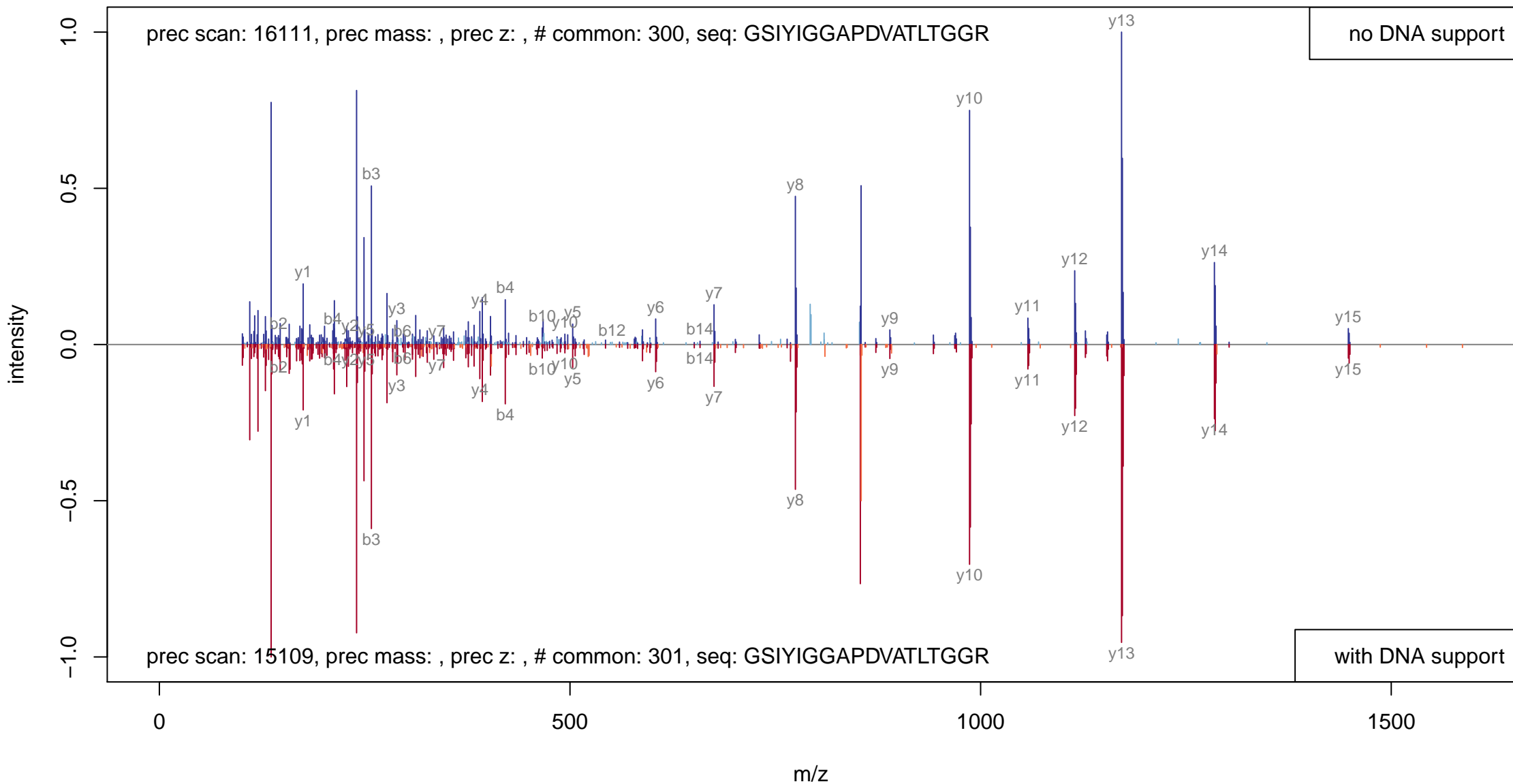

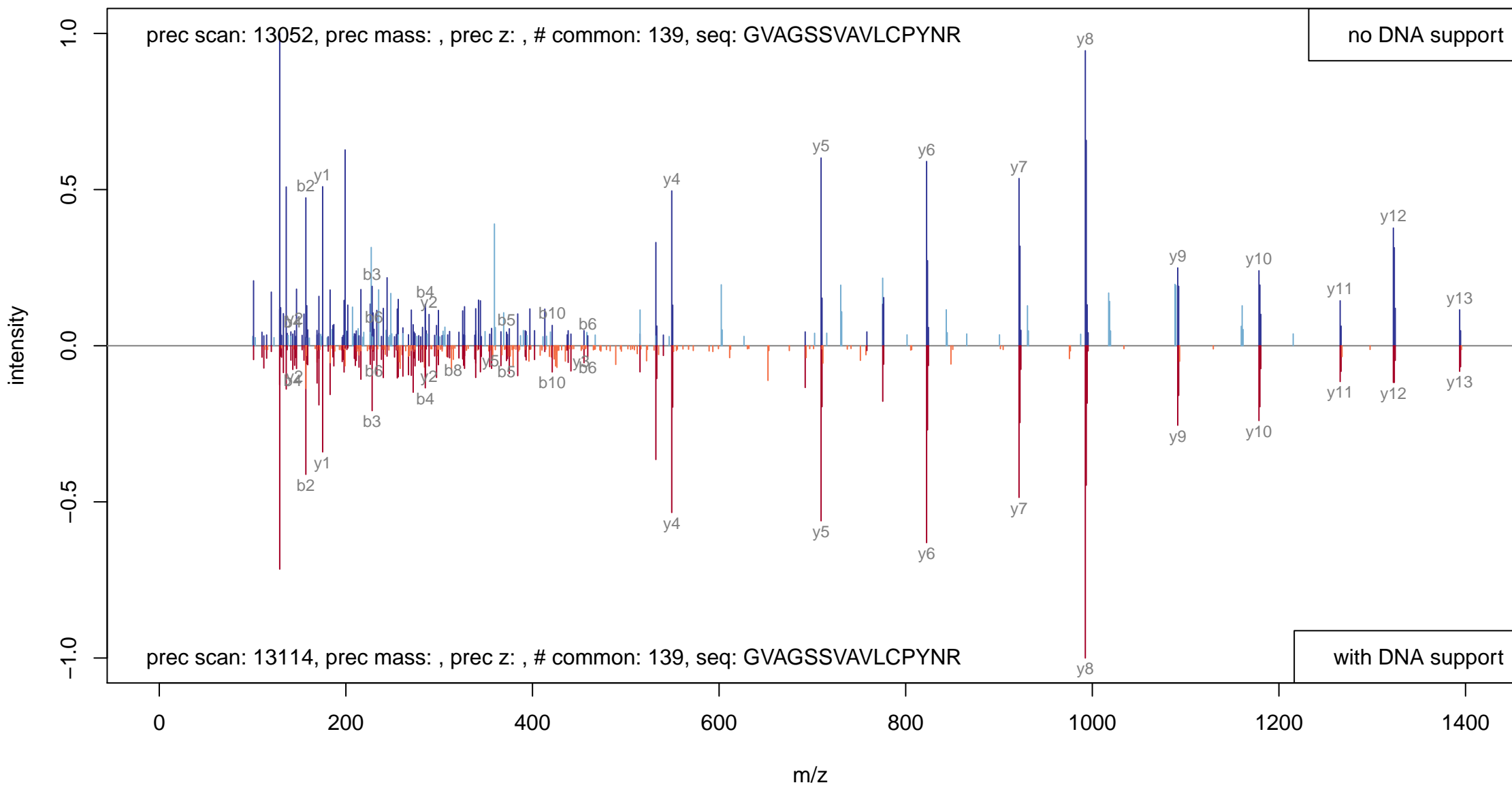

## Incorrect match

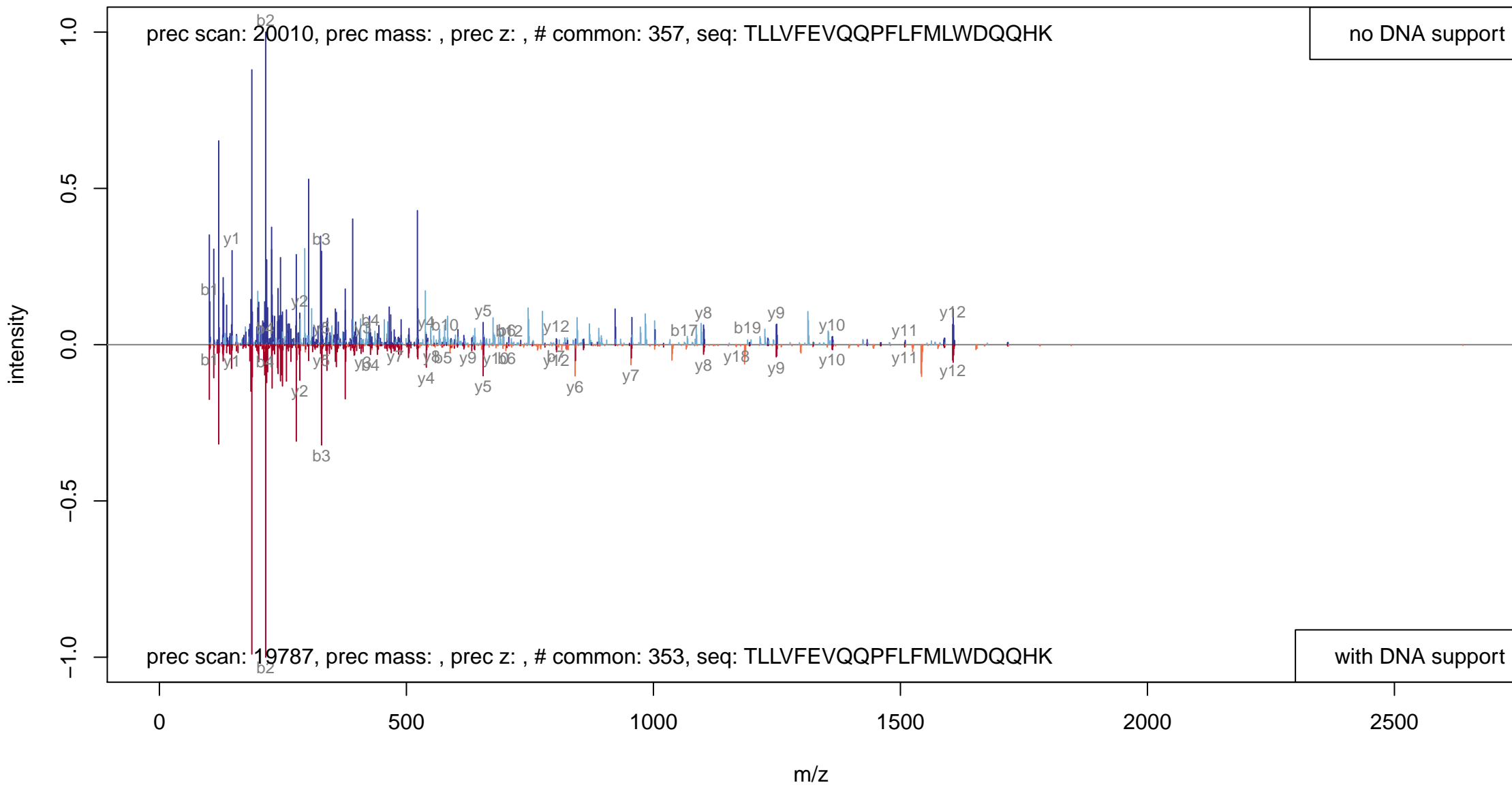

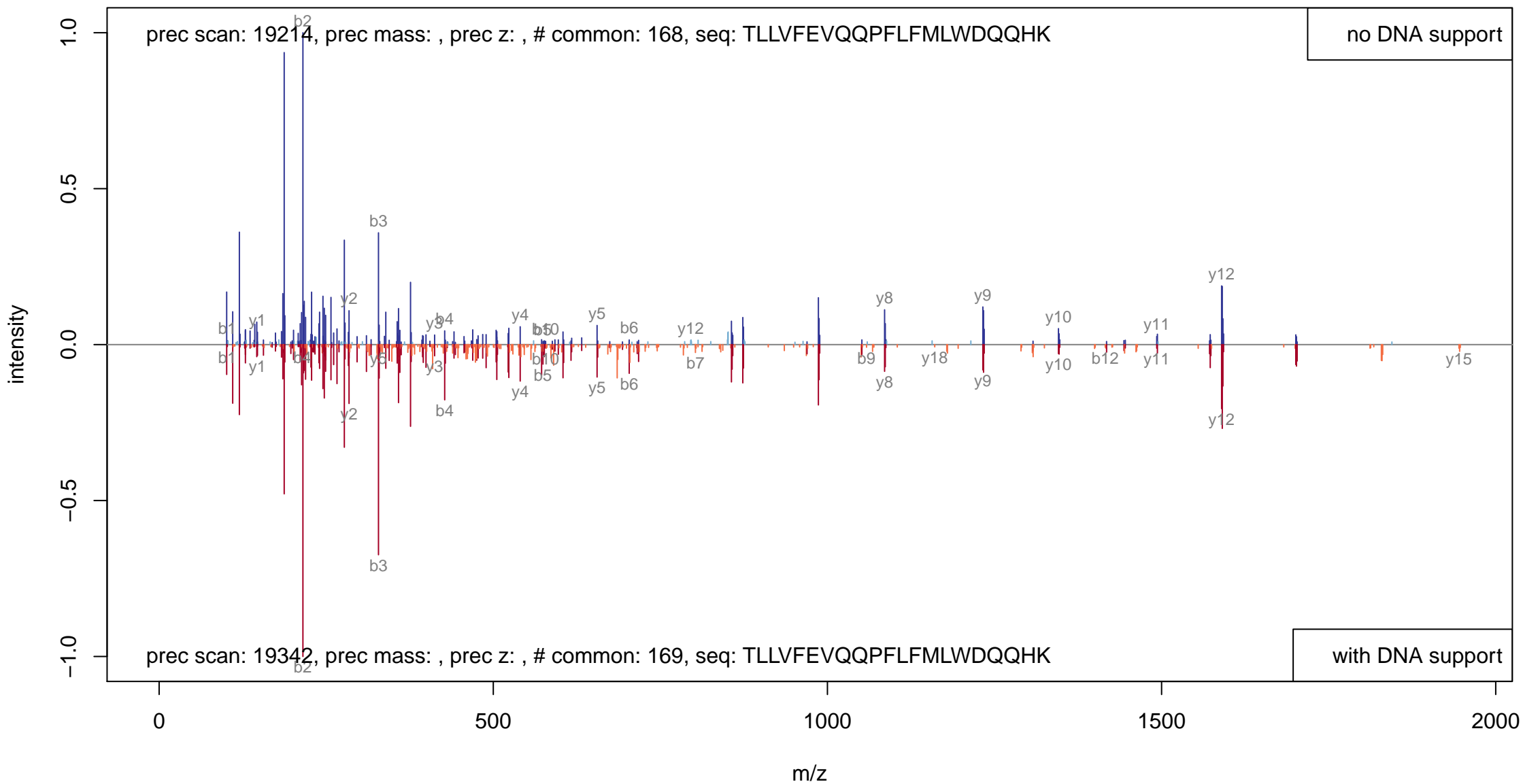

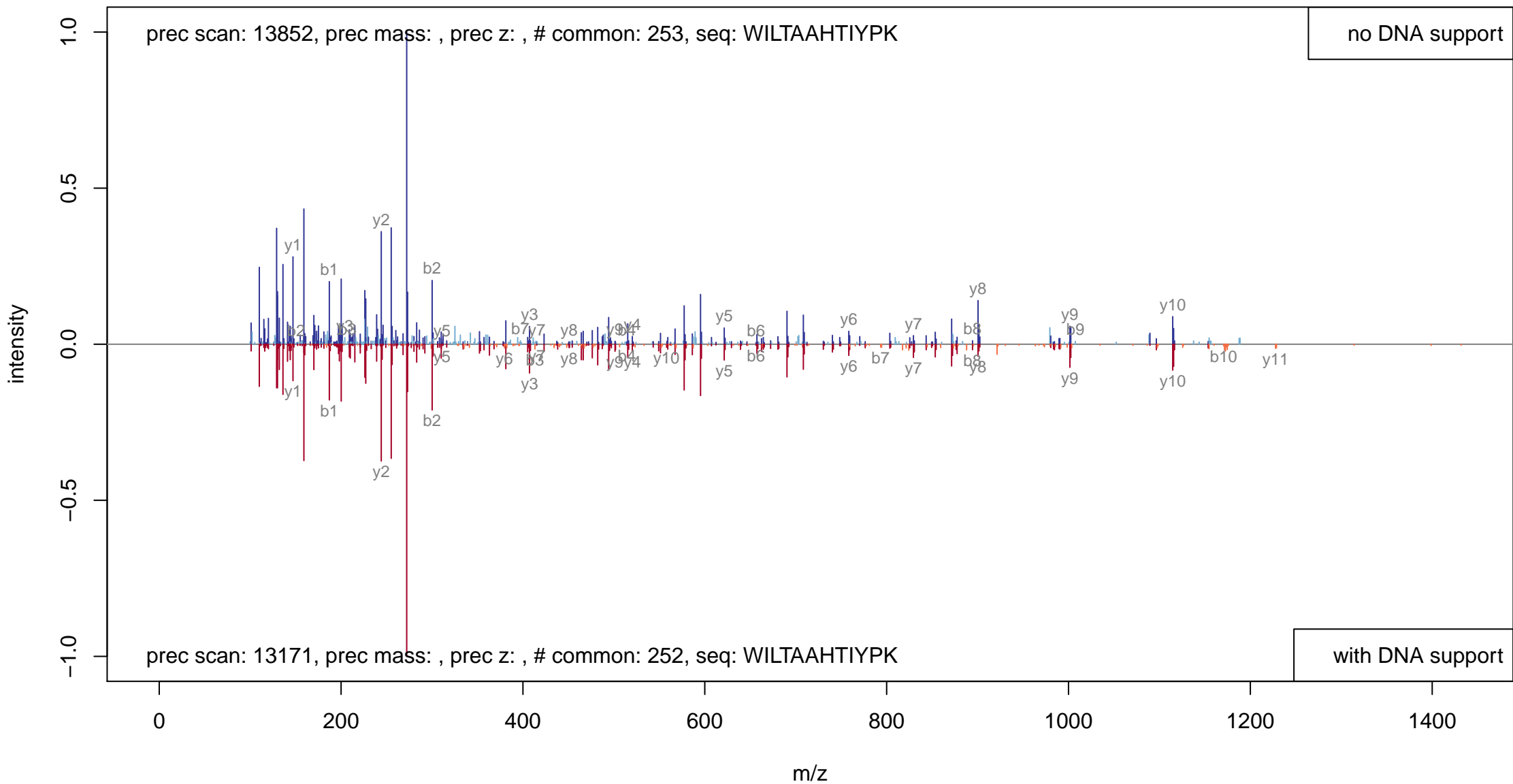

**incorrect match**

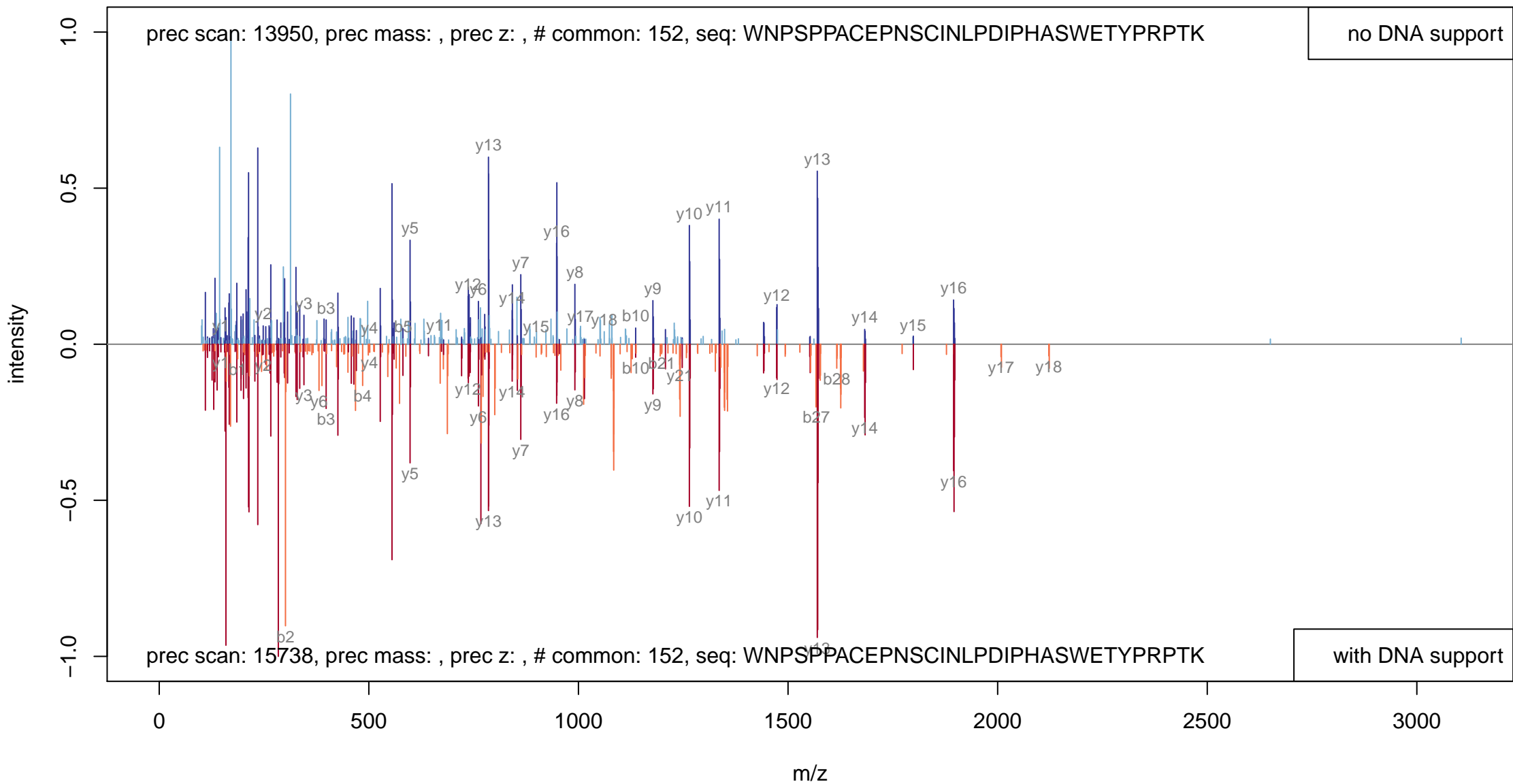

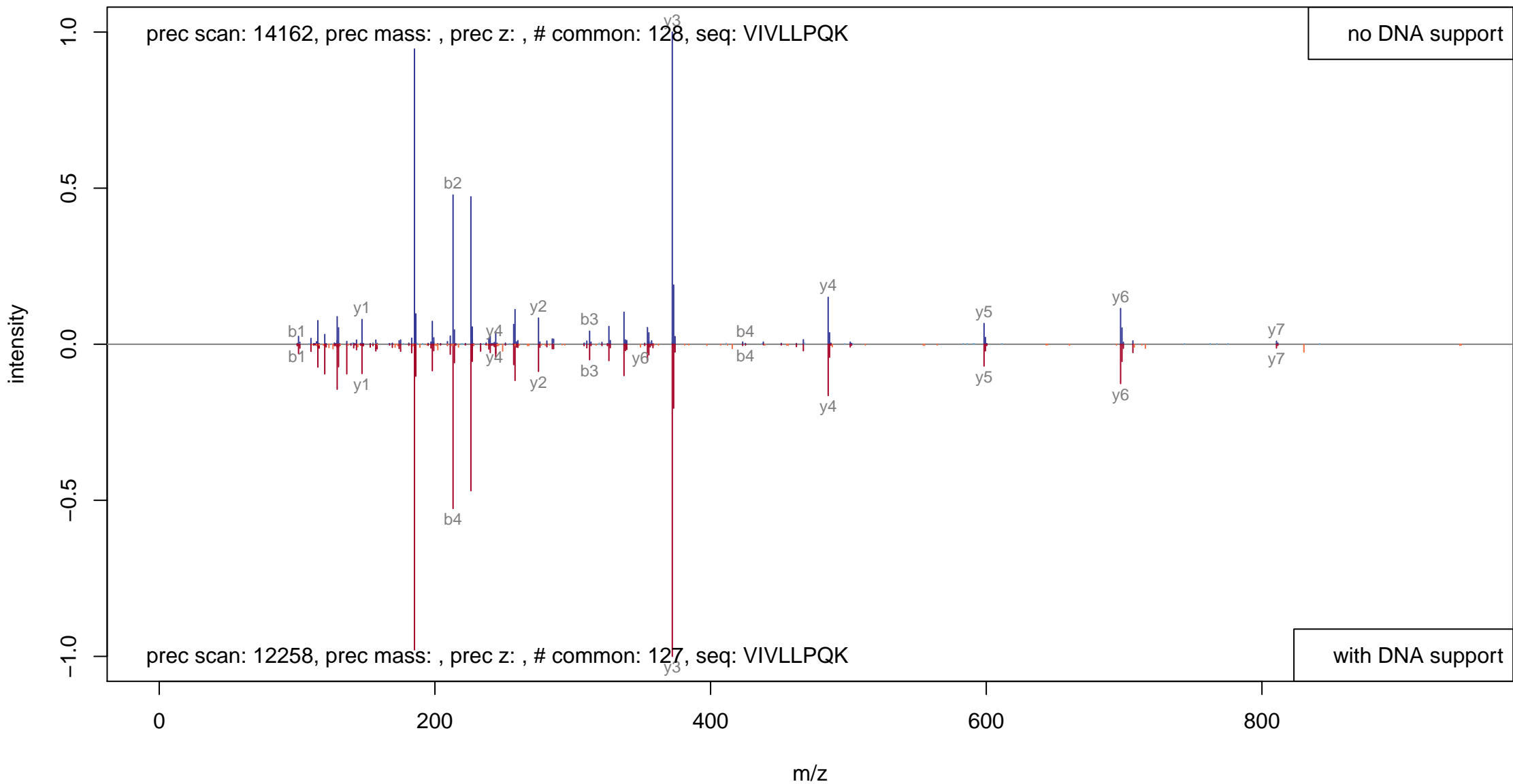

Supplement: Supplementary file 13. [file elife-41608-supp13.pdf]
